# Feedforward and feedback projections of caudal belt and parabelt areas of auditory cortex: refining the hierarchical model

**DOI:** 10.3389/fnins.2014.00072

**Published:** 2014-04-22

**Authors:** Troy A. Hackett, Lisa A. de la Mothe, Corrie R. Camalier, Arnaud Falchier, Peter Lakatos, Yoshinao Kajikawa, Charles E. Schroeder

**Affiliations:** ^1^Department of Hearing and Speech Sciences, Vanderbilt University School of MedicineNashville, TN, USA; ^2^Department of Psychology, Tennessee State UniversityNashville, TN, USA; ^3^Laboratory of Neuropsychology, National Institutes of Mental HealthBethesda, MD, USA; ^4^Cognitive Neuroscience and Schizophrenia Program, Nathan Kline InstituteOrangeburg, NY, USA; ^5^Department of Psychiatry, Columbia University College of Physicians and SurgeonsNew York, NY, USA

**Keywords:** connections, brain, monkey, functional organization, laminar, architecture, anatomy, laminar

## Abstract

Our working model of the primate auditory cortex recognizes three major regions (core, belt, parabelt), subdivided into thirteen areas. The connections between areas are topographically ordered in a manner consistent with information flow along two major anatomical axes: core-belt-parabelt and caudal-rostral. Remarkably, most of the connections supporting this model were revealed using retrograde tracing techniques. Little is known about laminar circuitry, as anterograde tracing of axon terminations has rarely been used. The purpose of the present study was to examine the laminar projections of three areas of auditory cortex, pursuant to analysis of all areas. The selected areas were: middle lateral belt (ML); caudomedial belt (CM); and caudal parabelt (CPB). Injections of anterograde tracers yielded data consistent with major features of our model, and also new findings that compel modifications. Results supporting the model were: (1) feedforward projection from ML and CM terminated in CPB; (2) feedforward projections from ML and CPB terminated in rostral areas of the belt and parabelt; and (3) feedback projections typified inputs to the core region from belt and parabelt. At odds with the model was the convergence of feedforward inputs into rostral medial belt from ML and CPB. This was unexpected since CPB is at a higher stage of the processing hierarchy, with mainly feedback projections to all other belt areas. Lastly, extending the model, feedforward projections from CM, ML, and CPB overlapped in the temporal parietal occipital area (TPO) in the superior temporal sulcus, indicating significant auditory influence on sensory processing in this region. The combined results refine our working model and highlight the need to complete studies of the laminar inputs to all areas of auditory cortex. Their documentation is essential for developing informed hypotheses about the neurophysiological influences of inputs to each layer and area.

## Introduction

The auditory cortex of primates is spread out over a large portion of the superior temporal gyrus (STG) and plane. Current models recognize 13 areas, grouped into three major regions (core, belt, parabelt). The identification and classification of areas and regions is based on interpretation of their neuroanatomical and neurophysiological profiles (Pandya et al., 1969; Pandya and Sanides, 1973; Burton and Jones, 1976; Jones and Burton, 1976; Imig et al., 1977; Fitzpatrick and Imig, 1980; Galaburda and Pandya, 1983; Cipolloni and Pandya, 1989; Morel and Kaas, 1992; Morel et al., 1993; Jones et al., 1995; Kosaki et al., 1997; Hackett et al., 1998; de la Mothe et al., 2006; Smiley et al., 2007; Hackett and de la Mothe, 2009). Among the most informative and distinguishing features of the three regions are the topographic patterns of connectivity within and between them. The three areas that comprise the core region are densely interconnected with about eight areas in the surrounding belt region. The belt areas have strong connections with parabelt region, which is currently divided into two areas. The core areas have only sparse connections with the parabelt.

On the basis of these connections, a regional hierarchy has been proposed in which information received by the core is sequentially processed by areas in the belt, and then the parabelt (Hackett et al., 1998). A second gradient has also been proposed along the caudal-rostral axis of the temporal lobe, based on the patterns of connections and architectonic gradients (Hackett, 2011). Although scant data are available, the known laminar projections suggest that information tends to flow from caudal to rostral areas in a feedforward manner (dominant inputs to layer 4), whereas projections from rostral onto caudal areas tend to exhibit feedback laminar profiles (dominant inputs to supragranular and/or infragranular layers, especially layer 1) (Rockland and Pandya, 1979; Felleman and Van Essen, 1991). There is also a bit of evidence that the most caudal auditory belt areas (caudomedial, CM; caudolateral, CL) direct some feedforward projections caudally toward auditory-related areas in the temporoparietal junction, such as Tpt (Fitzpatrick and Imig, 1980; Galaburda and Pandya, 1983; de la Mothe et al., 2006). Thus, information flow within the auditory cortex appears to move along two major anatomical axes: core-belt-parabelt and caudal-rostral. Correlated with these anatomical patterns are gradients in neuronal response properties. Frequency tuning bandwidth, response latencies and stimulus specificity generally increase along these axes, whereas temporal precision tends to decrease (Rauschecker et al., 1995, 1997; Rauschecker, 1998a,b; Rauschecker and Tian, 2004; Lakatos et al., 2005a; Bendor and Wang, 2008; Petkov et al., 2008; Kusmierek and Rauschecker, 2009; Kikuchi et al., 2010; Scott et al., 2011; Camalier et al., 2012; Kusmierek et al., 2012).

Beyond the confines of the auditory cortex, information from the belt and parabelt areas reaches multiple auditory-related areas distributed throughout the brain. (Tranel et al., 1988; Kosmal et al., 1997; Hackett et al., 1999; Romanski et al., 1999a,b; Cavada et al., 2000; Lewis and Van Essen, 2000; Falchier et al., 2002, 2009; Ghashghaei and Barbas, 2002; Lavenex et al., 2002; Petrides and Pandya, 2002; Yukie, 2002; Rockland and Ojima, 2003; Barbas, 2007; Smiley et al., 2007; Saleem et al., 2008, 2013; Markov et al., 2014). A rostrally-directed stream reaches targets in the temporal pole, ventral, rostral and medial prefrontal cortex, rostral cingulate, parahippocampal areas and the amygdala. A caudally-directed stream flows from the caudal belt and parabelt areas into the temporoparietal junction, posterior parietal and occipital regions (such as secondary visual cortex), caudal and dorsal prefrontal areas, dorsal cingulate and parahippocampal areas. Additional output streams flow laterally from the belt and parabelt regions to the upper bank of the superior temporal sulcus (STS) and medially into the insula and retroinsular areas within the lateral sulcus (Galaburda and Pandya, 1983; Hackett et al., 1998; de la Mothe et al., 2006; Smiley et al., 2007).

**Figure 1:**
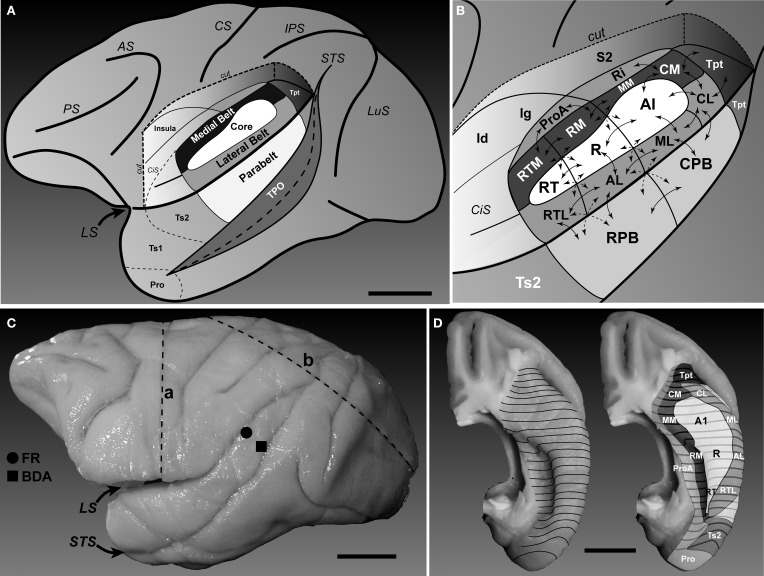
**Location and subdivisions of the auditory cortex in macaque monkeys**. **(A)** Lateral view of the left hemisphere showing position of auditory and auditory-related regions in the superior temporal region. The parabelt region is located on the surface of the superior temporal gyrus (STG). The core and belt regions lie on the superior temporal plane, visible after graphical removal of the overlying parietal and frontal opercula (cut). Area TPO lies on the upper bank of the superior temporal sulcus. The locations of the areas on the STG rostral to auditory cortex (Ts2, Ts1, and Pro) are also labeled (after Galaburda and Pandya, 1983). **(B)** Location and connections of areas within and around auditory cortex (see list of abbreviations for details). *Core region*: A1, R, RT; *Medial belt region*: CM, MM, RM, RTM; *Lateral belt region*: CL, ML, AL, RTL; *Parabelt region*: CPB, RPB; *auditory-related fields*, Tpt, Ri, Pro, S2, Ig, Id, Ts2. Arrows denote simplified patterns of connections between areas. Connections between non-adjacent areas not shown. **(C)** Photograph of the left hemisphere of Case 2 showing locations of injections (FR, filled circle; BDA, filled square) and blocking cuts (dashed lines a and b). Microtome sections were cut parallel to line b. **(D)** Dorsal views of the superior temporal plane showing locations of core, belt, and surrounding regions. The approximate locations of several areas are labeled. Caudal is up, lateral is right. Black contour lines run across the brain surface from medial (left) to lateral (right). Scale bars, 10 mm.

At present, the wiring diagrams that inform our models of auditory cortical function are low in resolution. The most widely used schematics depict the auditory areas on surface maps of the brain, using lines and arrows to denote a *connection* between one area and another (Figures 1A,B,D). These diagrams are a useful guide for describing the basic layout and connections of the auditory cortex, but reveal nothing about the laminar distributions of the somata, dendrites and axon terminals that comprise those connections and contribute to their functional importance (e.g., feedforward, feedback, etc.).

Unfortunately, these kinds of low-resolution maps reflect most of the current knowledge base. There are two reasons for this. First, most of the intrinsic and extrinsic connections identified in the experiments cited above used retrograde tracers, which permit identification of the input sources (neuronal somata) to the area targeted by a tracer injection. Relatively few of these studies used tracers with anterograde transport properties, which reveal the laminar projections from an area to its targets. Second, several studies utilized flattened brain preparations (cut parallel to the pial surface) for areal reconstruction of the patterns of labeled cells. While these methods are advantageous for creating surface maps of the connections between areas and regions, information about the laminar circuitry is not preserved. Fortunately, a few studies have been published in which the connections were studied in coronal sections using at least some tracers with anterograde transport properties (Fitzpatrick and Imig, 1980; Galaburda and Pandya, 1983; Aitkin et al., 1988; de la Mothe et al., 2006; de la Mothe et al., 2012). As noted above, sufficient data could be gleaned from these studies to support hypotheses about information flow along the two major axes (Hackett, 2011). However, these data are far from complete, derived from mixed primate species using different methods, and inconsistent at several levels of analysis. Lacking, and desperately needed, is an extended series of detailed anatomical studies in which anterograde and retrograde tracers are employed to more completely work out the laminar distribution patterns that actually comprise the connections between areas. Ideally, a survey of the input/output connections of each area should be obtained so that a complete wiring diagram of the auditory cortex could be made available. Such maps were developed over two decades ago for primate visual cortex (Felleman and Van Essen, 1991), and studies of both visual and somatosensory cortex have since progressed toward defining the connections of morphologically and neurochemically distinct neuronal subpopulations in every layer and sublayer (Callaway, 2002; Thomson and Bannister, 2003; Bannister, 2005; Douglas and Martin, 2007; Briggs, 2010; Feldmeyer et al., 2013). This implies that for primate auditory cortex, we are at the early stages of obtaining the kinds of basic data that were summarized over 20 years ago by researchers studying the visual cortex. This is an essential task, and one that will require sustained effort to complete.

With that in mind, the present study represents the beginning of what we intend to expand into a comprehensive accounting of the intrinsic connections of the primate auditory cortex. By injecting tracers with bidirectional transport properties in caudal belt (ML, CM) and parabelt (CPB) areas, our primary goals were to begin acquisition of the anatomical data, and at the same time test key predictions of our working model. For example, if the belt is driving activity in the parabelt, then projections from the belt should (at least) target layer 4 (L4) of the parabelt. Similarly, if caudal areas are driving rostral areas, then projections from the caudal areas should target L4 of the rostral areas. In addition, these experiments provided us with an opportunity to better characterize the nature of the inputs to the temporoparietal occipital area (TPO) on the upper bank of the STS, which is broadly connected with numerous auditory, visual, somatosensory, prefrontal, and posterior parietal areas. Earlier observations suggested that projections from the STG project to L4 and other layers of TPO (Cusick et al., 1995; Seltzer et al., 1996), indicating that the parabelt could be the main source of feedforward auditory input to this multisensory region.

## Materials and methods

Three macaque monkeys were used in this study (1 *macaca mulatta*, 2 *macaca radiata*). All procedures involving animals were conducted in accordance with international standards on animal welfare, followed NIH Guidelines for the Care and Use of Laboratory Animals, and were approved in advance by the Vanderbilt University Institutional Animal Care and Use Committee.

### General surgical procedures

Aseptic techniques were employed during all surgical procedures. Animals were premedicated with cefazolin (25 mg/kg), dexamethasone (2 mg/kg), and robinul (0.015 mg/kg). Anesthesia was induced by intramuscular injection of ketamine hydrochloride (10 mg/kg) then maintained by continuous isoflurane (2–3%) inhalation blended with 100% oxygen (1 L/min) through an endotracheal tube. Body temperature was held at 37°C with a water circulating heating pad. Heart rate, expiratory CO_2_, and O_2_ saturation were continuously monitored throughout the surgery and used to adjust anesthetic depth. For all surgical procedures, the head was held by hollow ear bars affixed to a stereotaxic frame (David Kopf Instruments, Tujunga, CA).

In Cases 1 and 3, injections of ML and CM were made using a vertical approach through chronic recording chambers (Crist Instruments, Hagerstown, MD) implanted over the auditory cortex in the left hemisphere. Injections were made through the injection ports of 24-channel linear array electrodes (U-Probe, Plexon Inc., Dallas, Tx) after completion of chronic electrophysiological recordings. The details of this procedure are explained in Smiley et al. (2007), where we first used the U-Probe for this purpose. Briefly, current source density analysis was used to initially position the deepest electrode channels between the pia and white matter. Slight adjustments in position are made to center the injection port at the prominent current sink in L4. The full volume of tracer (Table 1) was injected in 3 equal boluses, which disperses by capillary action up and down the electrode shaft, effectively depositing tracer across all cortical layers.

**Table 1:** **Experimental details for each case**.

**Case**	**Species**	**Sex**	**Areas injected**	**Method**	**Tracer**	**%**	**Volume (ul)**
1	*M. radiata*	M	ML	U-Probe	CTB	1	0.4
2	*M. radiata*	F	ML	Hamilton	10 kDa FR	10	0.8
			CPB		10 kDa BDA	10	0.5 × 2
3	*M. mulatta*	F	Rostral CM	U-Probe	10 kDA BDA	10	0.4

*Areas of tracer injections (ML, middle lateral belt; CM, caudal medial belt; CPB, caudal parabelt). Neuroanatomical tracers (CTB, cholera toxin subunit B; BDA, biotinylated dextran amine; FR, fluororuby). Aqueous concentrations and volumes injected are listed for each tracer*.

In Case 2, a midline incision was made exposing the skull, followed by retraction of the temporal muscle. A craniotomy was performed exposing the left dorsal STG, lateral fissure, and overlying parietal cortex. After retraction of the dura, warm sterilized silicone oil was applied to the brain to prevent desiccation of the cortex. Tracer injections were made into target areas through a pulled glass pipette affixed to a 1 μ l Hamilton syringe. The pipette was advanced into cortex under stereo microscopic observation to a depth of 1000 μm using a stereotaxic micromanipulator. After manual pressure injection of tracer into each target area (Table 1), the syringe was held in place for 10 min under continuous observation to maximize uptake and minimize leakage. Injection the CPB were made directly into the lateral surface of the STG after removal of the dura. Injection of ML was achieved by slight retraction of the banks of the lateral fissure, as previously described (Hackett et al., 2005).

### Tracer injections

In all cases, tracers were injected by pressure into target areas of the auditory cortex. Table 1 contains the relevant experimental details of each case, including tracer type, tracer volume, injection device, and area injected. In Cases 1 and 3, tracer injections were made subsequent to a series of electrophysiological recordings to more accurately identify the target area and surrounding areas. In Case 1, the injection into area ML was made through the recording chamber during a routine awake-behaving recording session. Recordings in this case broadly covered 10 areas of auditory cortex, as reported in Camalier et al. (2012). In Case 3, the injection into the rostral-medial limb of area CM was made through an established recording chamber following recordings focused on areas A1 and CM. Spatial mapping density was not dense in either of these cases, and did not significantly interfere with tracer transport or architectonic assays. In some figures, electrode tracks or lesions can be seen and these are marked with asterisks. In Case 2, the injections into areas ML (near its the caudal and lateral borders) and CPB (caudal and ventral quadrant) were made directly into cortex through a craniotomy under general anesthesia (see above) and in the absence of electrophysiology. Stereotaxic coordinates and surface landmarks were used to identify the target locations. In an attempt to avoid involving the dorsal CPB, the ML injection was made medial to the middle cerebral vein, by slight retraction of the dorsal bank of the lateral sulcus (Hackett et al., 2005). Note that the proximity of the injection to the CPB border (Figure 4E). The spread of tracer was minimal, but may have encroached slightly into the CPB, and if so, mainly in L1-3a. Although the injection obscured architectonic features and absolute confirmation, the dense retrograde labeling in A1 is consistent with a significant deposit in ML, since CPB injections rarely produce labeled cells in A1 (Supplementary Figure 2 and Supplementary Table 2). If the FR tracer was transported by any cells in the dorsal CPB, we are unable to determine that from the labeling patterns observed.

Three tracers were used in these studies: cholera toxin subunit B (CTB) (Vector Labs); 10 kDa tetramethylrhodamine (aka fluororuby, abbreviated as FR) (Molecular Probes); 10 kDa biotinylated dextran amine (BDA) (Sigma). FR and BDA have the potential for bidirectional axonal transport (anterograde and retrograde), but are most sensitive as anterograde tracers and produce well-defined labeling of axon arbors and terminal puncta. CTB is a very sensitive retrograde tracer, but uptake often produces strong anterograde transport, as in Case 1 and our previous studies in marmosets (de la Mothe et al., 2006). Rather than punctate labeling of terminals and their axonal arbors, anterograde transport of CTB typically produces a “dust-like” deposit in the terminal zone intermingled with some punctate terminal labeling. This labeling is fine enough to be localized to specific to laminae and sublaminae, but the contacts of individual terminal puncta cannot usually be resolved.

### Perfusion and histology

After a 14–21 days survival period, a lethal dose of pentobarbital (120 mg/kg) was administered intravenously. Just after cardiac arrest the animal was perfused through the heart with cold (4°C) saline, followed by cold (4°C) 4% paraformaldehyde dissolved in 0.1 M phosphate buffer. Following perfusion the brains were removed and photographed. The cerebral hemispheres were blocked and placed in 30% sucrose for 3 days. The cerebral hemispheres of each case were cut at slightly different angles. In Cases 1 and 3, the angle was very close to coronal, matching the angle of electrode penetrations through the recording chamber. In case 2, the angle was perpendicular to the lateral sulcus in the caudal to rostral direction at 40 μm, as shown in Figure 1C (line “b”). This minimized cross-cutting across cortical columns for areas in the lateral, superior temporal, inferior parietal, and central sulci.

In each brain, series of 12 sections were alternately processed for the following set of histochemical markers: (i) fluorescent tracer microscopy; (ii) biotinylated dextran amine (BDA) or cholera toxin subunit B (CTB); (iii) acetylcholinesterase (AChE) (Geneser-Jensen and Blackstad, 1971); (iv) stained for Nissl substance with thionin. Additional reactions were performed in some cases to facilitate reconstruction, or obtain data for other studies: (v) cytochrome oxidase (Wong-Riley, 1979); (vi) parvalbumin (PV); (vii) vesicular glutamate transporters 1 (VGluT1) and 2 (VGluT2) (Hackett and de la Mothe, 2009); (ix) neuron specific nuclear protein, NeuN (Hackett and de la Mothe, 2009), and (x) myelinated fibers (MF) (Gallyas, 1979). In cases 2 and 3, multifluorescent immunohistochemistry (IHC) of NeuN and VGluT2, VGluT1 or PV were combined with fluorescent detection of tracers in single sections to relate areal and laminar boundaries to the locations of axon terminals and somata. Auditory areas were identified using these markers according to detailed architectonic criteria established in previous studies of macaque monkeys (Hackett et al., 2001; Smiley et al., 2007; Hackett and de la Mothe, 2009), and also applied to marmoset monkeys (de la Mothe et al., 2006). In brief, the cytoarchitecture of the core areas stands out for a broad densely packed L4, separated from L6 by a cell-sparse L5. The middle layers of the core stain darkly for PV, VGluT2, MF, and AChE compared to sharp reductions in density at borders with adjacent belt areas. The parabelt transition from lateral belt to parabelt is usually within 1 mm of the edge of the lateral sulcus. It is not always sharply demarcated, but characterized by a reduction in L4 density of these markers. There is a gradual reduction in the prominence of these markers from along the caudal-to-rostral axis, which is used to distinguish adjacent areas along that axis. The border between parabelt and TPO is generally near the lateral edge of the STS, and its precise location also appears to meander somewhat along the rostral-caudal axis. Supplementary Figures 1A,B shows an example of fluorescent NeuN IHC paired with an adjacent section stained for VGluT2 and identification of areas in a coronal section through A1. The panels below illustrate how laminar boundaries were located in sections containing FR and BDA tracers in three different areas (C–F, area ML; G–J, area MM, K–N, area TPO).

### Microscopy and reconstruction of sections

**Figure 2:**
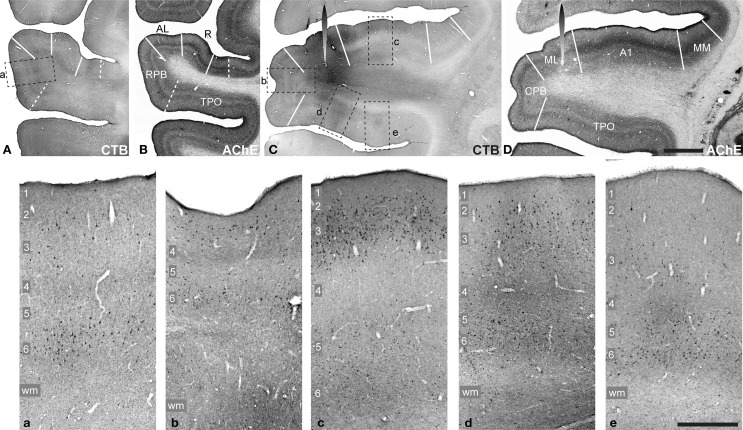
**CTB labeling (A,C, a–e) and AChE histochemistry (B,D) in Case 1**. CTB injection into area ML visible in **(C,D)** (spearhead symbol). Adjacent sections processed for CTB and AChE at a more rostral location at the level of the RPB **(A,B)**. Rectangles in **(A,C)** denote locations of panels **a–e**, below. These panels contain higher magnification images from the RPB **(a)**, CPB **(b)**, A1 **(c)**, and TPO **(d,e)** to show retrograde and anterograde transport in layers of each cortical area. Scale bars: **(A–D)**, 2 mm; **a–e**, 500 μm.

**Figure 3:**
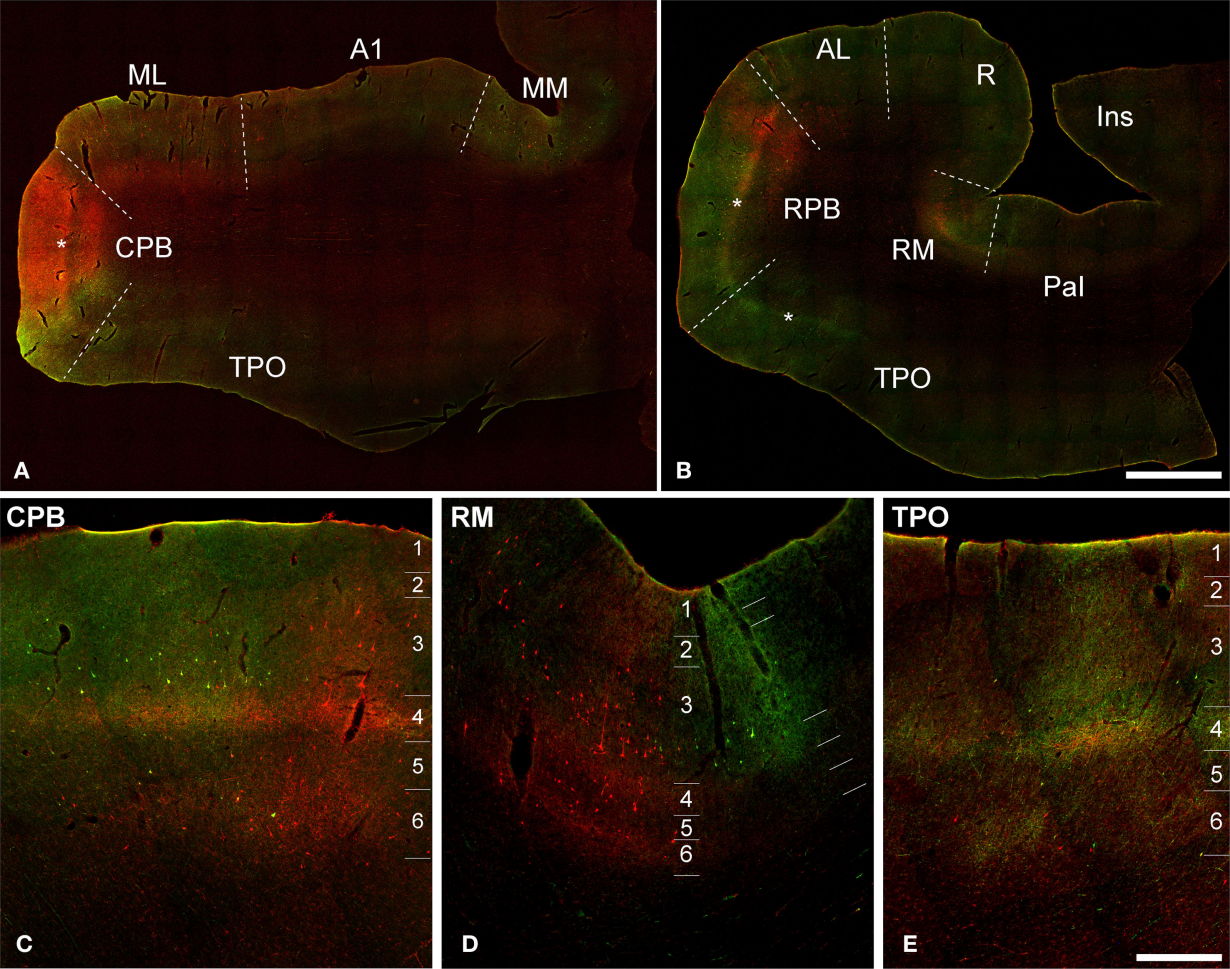
**Dual fluorescence images of BDA (green) and FR (red) labeling of axons, terminals, and somata in Case 2**. **(A,B)** Low magnification sections from caudal **(A)** and rostral **(B)** sections. Asterisks denote position of layer 4. **(C–E)** Higher magnification views through portions of CPB, RM, and TPO show overlapping BDA and FR signals. Note overlapping bands in L4 and L6 from both tracers, and patchy columnar labeling across layers in all panels. Scale bars: **(A,B)** 2 mm; **(C–E)** 500 μm.

**Figure 4:**
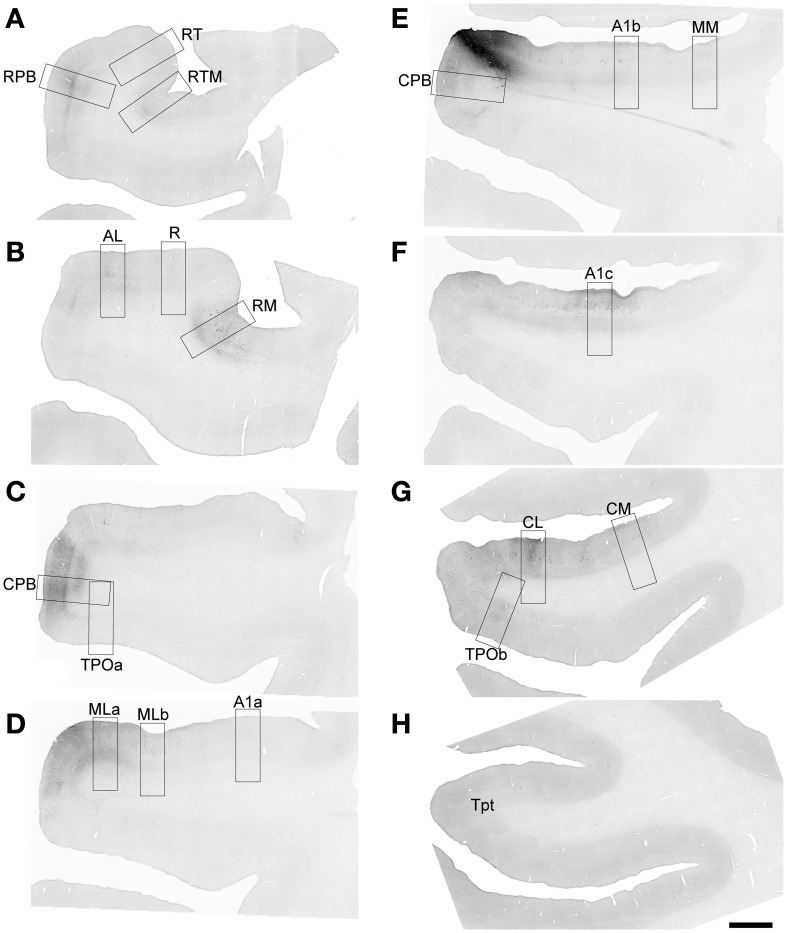
**FR labeling by the injection of ML in Case 2**. Low magnification images of sections oriented from rostral **(A)** to caudal **(H)** along the STG. Black signal visible in each panel shows anterograde and retrograde transport from the FR injection in the caudal and lateral portions of ML **(E)**. Rectangular selections correspond to higher magnification images in Figure 5. Scale, 2 mm.

**Figure 5:**
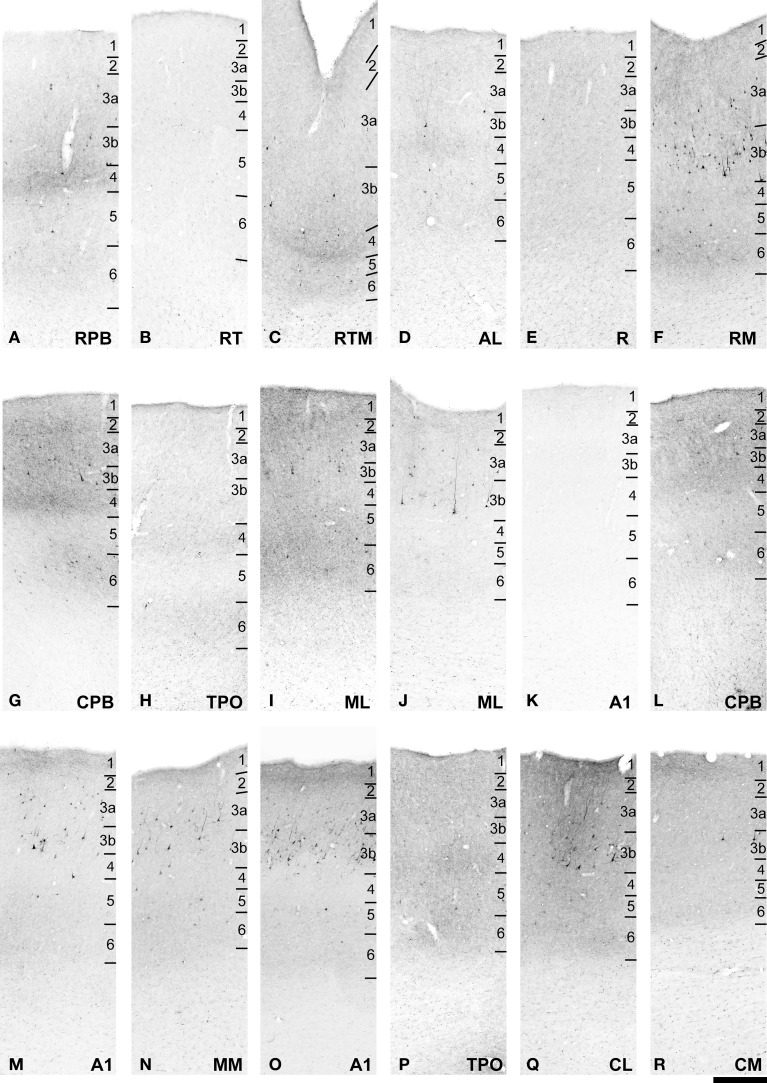
**FR labeling of areas corresponding to rectangular selections in Figure 4**. **(A–K)** from **(A–D)** in Figure 4. **(L–R)** from **(E–H)** in Figure 4. See text for detailed descriptions and Figure 10 for graphical summary of these data. Scale, 500 μm.

**Figure 6:**
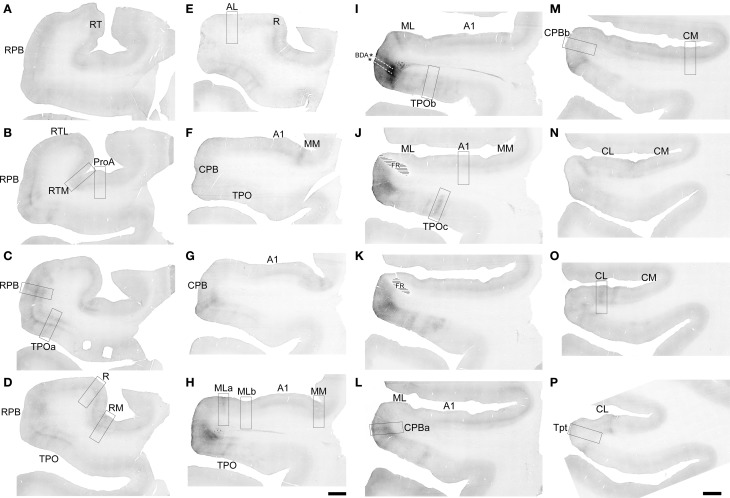
**BDA labeling by the injection of CPB in Case 2**. Low magnification images of sections oriented from rostral **(A)** to caudal **(P)** along the STG. Black signal visible in each panel shows anterograde and retrograde transport from the BDA injection in the caudal and ventral portions of CPB **(I)**. Rectangular selections correspond to higher magnification images in Figure 7. Location of FR injection indicated by hatching in **(J,K)**. Scale, 2 mm.

**Figure 7:**
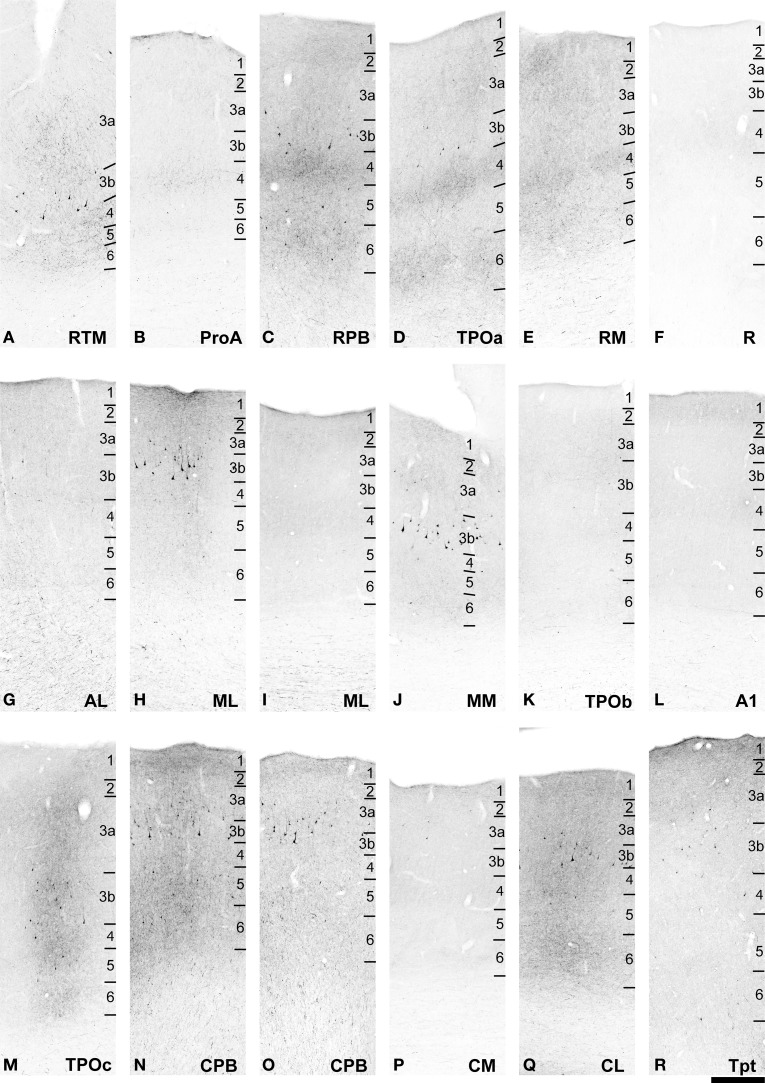
**BDA labeling of areas corresponding to rectangular selections in Figure 6. (A–J)** from **(A–H)** in Figure 6. **(K–R)** from **(I–P)** in Figure 6. See text for detailed descriptions and Figure 10 for graphical summary of these data. Scale, 500 μm.

**Figure 8:**
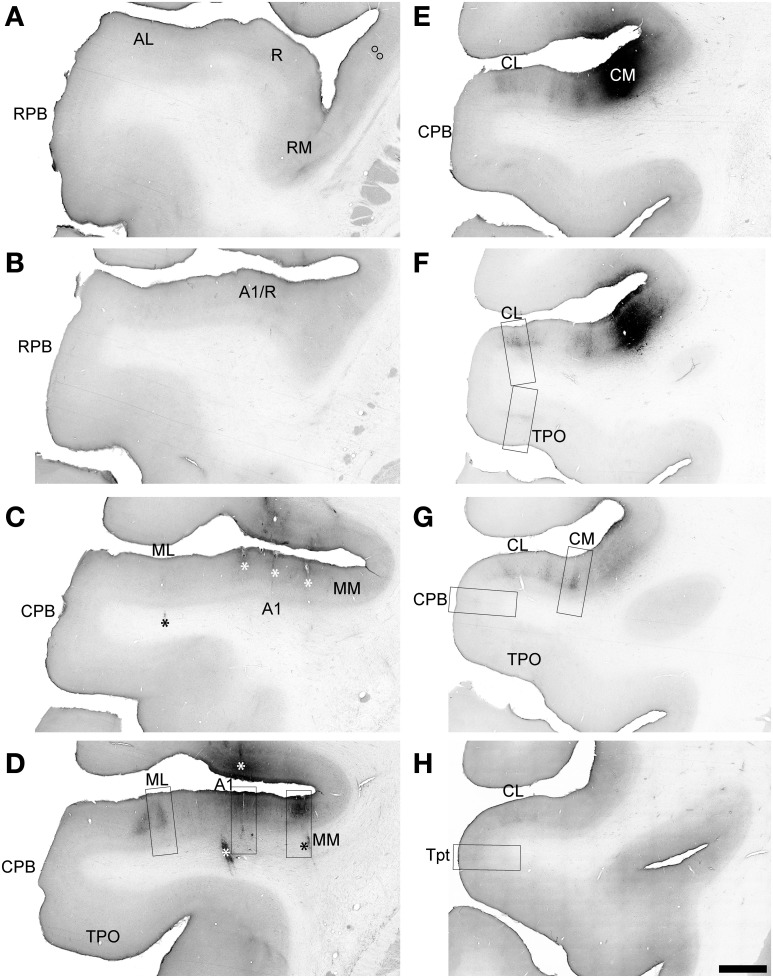
**BDA labeling by injection of CM in Case 3**. Low magnification images of sections oriented from rostral **(A)** to caudal **(H)** along the STG. Black signal visible in each panel shows anterograde and retrograde transport from the BDA injection in the rostral and medial portions of CM **(E,F)**. Rectangular selections correspond to higher magnification images in Figure 9. Open symbols in **(A)** indicate location of labeled cell somata in the insula. Asterisks in **(C,D)** denote locations of electrolytic lesions. Scale, 2 mm.

**Figure 9:**
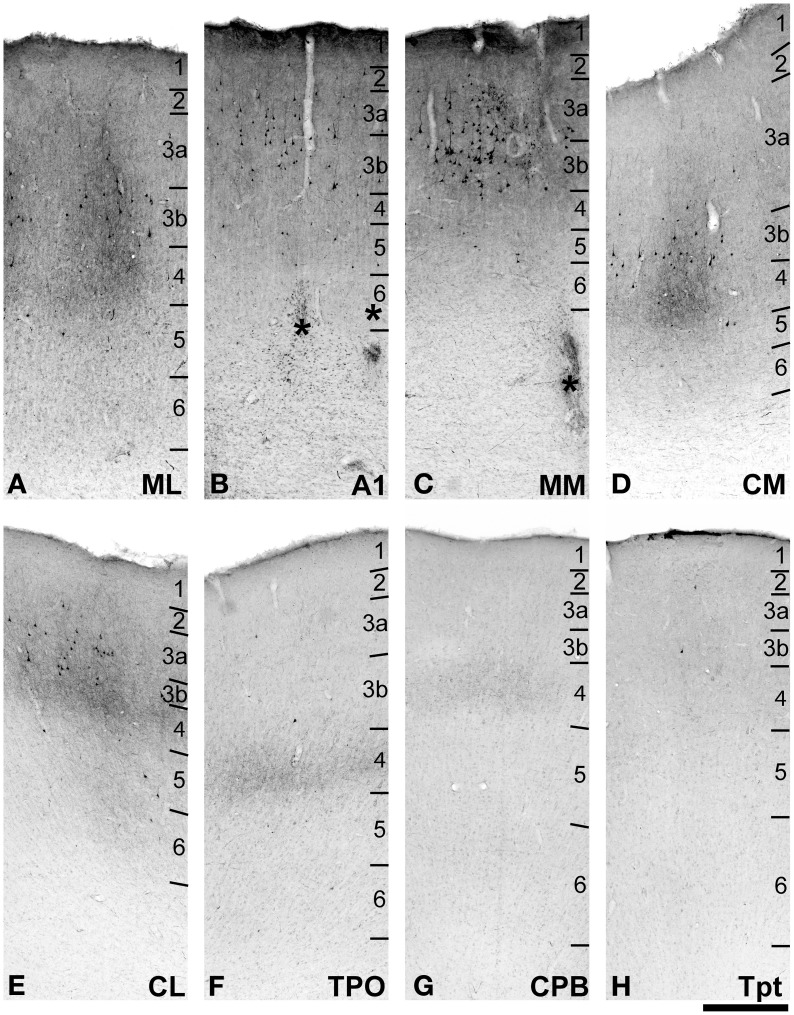
**BDA labeling of areas corresponding to rectangular selections **(A–H)** from **(D–H)** in Figure 8**. Asterisks in **(B,C)** denote locations of electrolytic lesions. See text for detailed descriptions and Figure 10 for graphical summary of these data. Scale, 500 μm.

**Figure 10:**
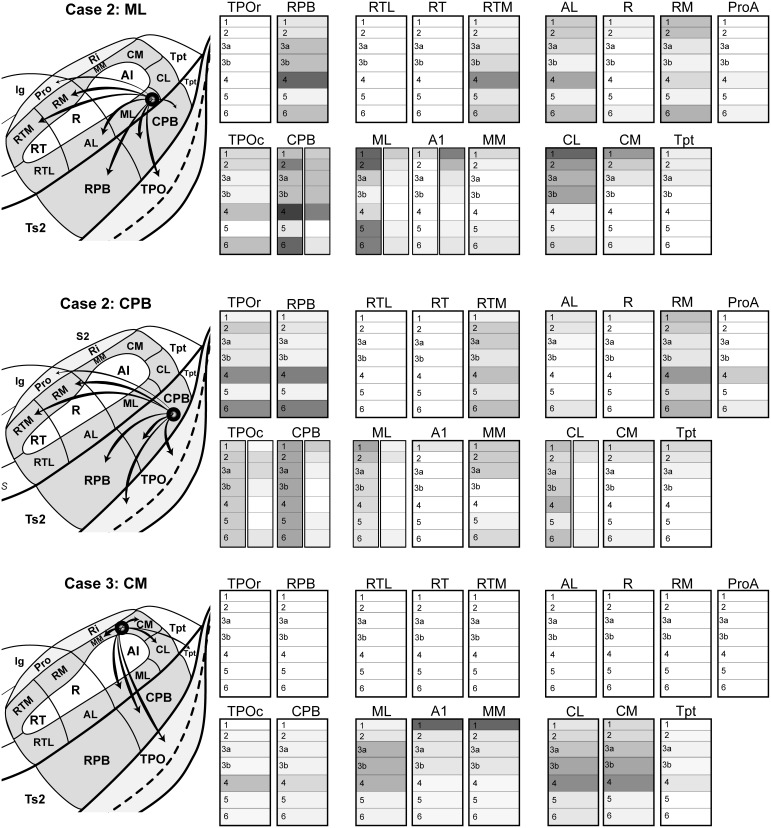
**Summary of areal and laminar projection profiles from Cases 2 and 3, derived from Figures 4–9 and Supplementary Table 2**. *Left*, schematics of the auditory cortex and TPO showing the principal feedforward projections from the location of the injection site in each case: *Case 2 ML*, FR injection into caudal-lateral ML; *Case 2 CPB*, BDA injection into caudal-ventral CPB; *Case 3 CM*, BDA injection into rostral-medial CM. *Right*, for each cortical area, the density of anterograde terminal labeling is represented by layer. For some areas (CPB, ML, TPO), two laminar profiles are illustrated side-by-side to depict laminar patterns in different parts of the same area. Dark and light shading denote high and low density. Absence of shading (white) denotes little to no transport to this area or layer.

Digital images of brightfield sections were acquired using a Neurolucida system (MicroBright Field, Inc., Williston, VT) and Nikon 80i microscope. Fluorescent images were acquired using a Hamamatsu Orca digital camera and Nikon 90i microscope. All of the images in Figures 2–9 are photomontages stitched from a matrix of multiple photographs obtained using a 10× objective and Nikon Elements AR software. These images were cropped, adjusted for brightness and contrast using Adobe Photoshop CS6 software. Images of sections containing transported tracers were selected at regular intervals (~1:24) for illustration in rostral-to-caudal sequence. Final figures containing images and line drawings were made using Adobe Illustrator CS6 (Adobe Systems, Inc.). All descriptions of anterograde tracer deposits recorded in the text and figures were based on calculation of relative optical density in each layer of each cortical area. Digitized images of each cortical area (see Figures 5, 7, 9) were converted to 8-bit grayscale images and imported into ImageJ at full resolution (Rasband, W.S., ImageJ, U. S. National Institutes of Health, Bethesda, Maryland, USA, http://imagej.nih.gov/ij/, 1997–2014). For each cortical area, inverted grayscale levels (GL) from 0 (white) to 255 (black) were measured in each layer from regions of interest encompassing labeled terminals drawn using the polygon tool, avoiding artifacts, blood vessels, and retrogradely labeled cells. Background grayscale levels (BL) were measured from a separate region devoid of terminals in the white matter just below layer 6. The Gray Level Index (GLI) was calculated as follows: GLI = (GL–BL)/BL. These values are recorded in Supplementary Table 1, and used to set the intensity of layers in each panel of Figure 10. A “feedforward” (FF) connection type was defined as an axonal projection that produced concentrated terminal labeling in L4. Almost invariably, feedforward inputs to L4 of a recipient area were accompanied by band of labeled axons and terminals concentrated in other layers, most often L1 and L6. This was sometimes accompanied by weaker axon and terminal labeling in the intervening layers (L2–3B, L5). We refer to these projections as “lateral” connections. Projections that were concentrated in L1 or L1 and L6 are referred to as “feedback.” These designations are based on prior studies (Rockland and Pandya, 1979; Felleman and Van Essen, 1991).

## Results

### Case 1: ML injection

The sentinel case, which initiated the present study, was Case 1 (Figure 2). An injection of CTB was placed into ML, as part of an ongoing study of retrograde transport to the thalamus. Figure 2 contains images of tissue sections at the level of ML, containing the injection site (C,D), and more rostral sections at the level of the RPB (A,B). At each level, sections stained for CTB (A,C) are depicted with a nearby section stained for AChE (B,D), as an example of the architecture that was used to identify areas of auditory cortex. In the panels below (a–e), higher magnification images from the RPB (a), CPB (b), A1 (c), and TPO (d,e) show retrograde and anterograde transport across laminae.

The injection spanned all cortical layers and also appeared to involve the white matter directly below layer 6. Transport from this injection produced retrograde labeling of somata in nearly all areas of auditory cortex and ventrally across much of TPO on the upper bank of the STS. In addition, although CTB does not always produce detectable anterograde labeling, this case was an exception, as anterograde deposits were reliably present in areas where retrogradely labeled cells were found, and also in laminae (e.g., L1, L4) where they were not. Of particular interest was that anterograde transport was present in L4 and L6 of the RPB and CPB (Figures 2Aa,Cb), and extended across most of TPO (Figure 2Cd,e). Dense patches of overlapping anterograde and retrograde labeling, sometimes spanning all layers, were also present in TPO (d,e). In contrast, anterograde labeling in L4 of A1 was largely absent (c), despite strong anterograde and retrograde labeling in supragranular and infragranular layers. These laminar profiles suggested that feedforward and lateral projections from ML targeted RPB, CPB, and TPO, whereas projections to A1 were a type of feedback projection.

Further analyses of cortical transport were not pursued in this case, because of uncertainty about the quality of anterograde CTB transport, and some concern that the involvement of the white matter by the injection may have been taken up by fibers of passage to or from the caudal parabelt. However, as the overall projection patterns were highly similar to those observed in subsequent cases, the data have supplementary value. Further, as CTB is a highly sensitive retrograde tracer, this case served as an important control for the weaker retrograde transport associated with the BDA and FR tracers noted in the other cases.

### Case 2: ML and CPB injections

The FR and BDA tracers used in this study produced both retrograde labeling of neuronal somata and punctate anterograde labeling of axons and terminals. Typically, labeled terminals were distributed regularly along axon segments, often oriented horizontally (parallel to pial surface and laminae), crossing several cell columns. Some of these axons could be followed for several millimeters, and tended to be more common in L6 and white matter. Diagonal and vertical (radial) orientations were less common, and could occur in any layer. Occasionally, a radially-oriented axon traversed multiple layers, spanning infra and supragranular domains.

Figure 3 depicts sections at different magnification from Case 2, in which BDA-labeling from injection of CPB was made fluorescent by reacting the sections with streptavidin tagged with a green fluorescent marker (AlexaFluor 488). This allowed simultaneous viewing of terminals from the FR injection of ML (red) and BDA injection of CPB in the same section (NeuN fluorescence for laminar identification not illustrated, but see Supplementary Figure 1). In panels A and B, sections from caudal (at level of A1) and rostral (at level of R) locations were selected for illustration. The panels below (C–E) are higher magnification views through portions of CPB, RM, and TPO where overlapping signals from both tracers are clearly visible. These panels reveal zones of overlapping and non-overlapping transport of FR and BDA to L3 and L4 of these areas. These results illustrate the expected topographic differences between two different areas of auditory cortex, but also reveal a high degree of overlap in L4 and L6, for example, which implies that both input sources (ML and CPB) impact activity in the same cortical columns.

Note that, as in Figure 2, anterograde tracer deposits were dense enough in some locations that their laminar distributions were easily discerned even in low magnification images. Otherwise, labeled terminals and axons could only be resolved and visualized at higher magnification. In the figures of cases 2 and 3 that follow (Figures 4–9), anterograde terminal labeling is illustrated at lower and higher magnification for visualization of the details most relevant to this study Graphical representations of the projection patterns for each injection were also prepared to summarize this aspect of the results (Figure 10). Plots and cell counts of retrograde transport in this case are summarized in Supplementary Figure 2 and Supplementary Table 2.

#### Projections of area ML

The principal outputs of caudal ML reached core, belt, and parabelt areas along the entire rostral-caudal extent of the AC (Figures 4, 5, 10). Terminal labeling differed between areas and between layers. The strongest projections targeted areas at the same rostrocaudal level as the injection and areas just rostral to it.

Rostral to the injection, the most prominent projections from ML reached RPB, AL, RM, RTM and ProA (Figures 4A–D, 5A,C,F). These were characterized by lateral and feedforward style inputs, usually with foci in L4 and L6, which stood out from somewhat reduced density in other layers. Most surprising were the strong projections to RM and RTM, as these areas were not expected to be important targets of feedforward projections by ML. These inputs spanned all layers, with prominent foci in L4 and L6 that extended medially into Pro in the floor of the circular sulcus. Weaker projections also reached the rostral core area, R, and rostral A1 where a few branching axons were located in L1/2 and L6. Otherwise, in A1 (Figure 5K), labeled terminals extended in L1/2 across the entire width of field from ML to MM. There was no significant input to RT or rostral TPO, and very sparse inputs to L1 of RTL.

At the approximate rostrocaudal position of the injection (Figure 4E), lateral connections with A1 were most dense in the L1/2 band (Figures 5M,O), moderate in the layers beneath, and sparse in L4. In MM, terminals were mainly found in bands spanning L1/2 and L5/6 (Figure 5N).

Caudal to the injection, terminal labeling was found in CL, CM, CPB, Tpt, and TPO (Figures 4F–H). The strongest labeling in auditory cortex was in CL, where patches of dense terminal labeling extending across layers were bounded by patches of lesser labeling in L1–3b and L5–L6 (Figure 5Q). Light terminal labeling was found in L4. The laminar distributions in the intervening patches were comparable, but density was reduced overall across layers. In CM, labeling was patchy, as in CL, but not as dense (Figure 5R). In some ways, the laminar patterns in CM resembled those of A1, with heaviest concentrations in L1. Unlike A1, however, was moderate terminal labeling that extended across L2 and L3a, then became very sparse in L3b–6. In CPB caudal to the ML injection, terminal labeling was moderate in supra- and infragranular layers, but avoided L3b and L4. In Tpt, very light terminal labeling, continuous with CL and CPB, persisted onto its gyral and planar domains, but was primarily restricted to axonal branching in L1–3a. Retrogradely labeled cells were mainly located in L3b of CL and CM, with some neurons in L3a and L5/6 of these areas. These results indicate that caudally-directed projections from ML reach the caudal belt areas and Tpt, and are clearly biased toward the supragranular layers (L1–3b). Terminal labeling in L4 was light in CL, sparse in CM, and absent from Tpt, indicating that caudally-directed feedforward projections from ML are extremely limited. The retrograde labeling suggests that ML may be a recipient of feedforward projections from CL and CM, however.

Projections from ML to TPO on the upper bank of the STS were primarily confined to its caudal half (Figure 4G), and roughly in line with the rostral-caudal span of the CPB. These inputs were characterized by two basic laminar patterns, often visible in the same section: (1) full columns of labeled terminals that spanned all layers (Figure 5P), or (2) terminal foci in L1/2, L4, and L6 with very sparse label in the intervening layers. This laminar pattern often occurred in the zones, or patches, that separated those with the columnar labeling pattern. In rostral sections, in line with the RPB, light terminal labeling continued from the ventral RPB for a short distance into the lateral edge of TPO before ceasing entirely. Therefore, ML has direct lateral and feedforward projections to mainly the caudal sector of TPO that bypass the CPB.

Two other patterns of interest concerned the projections to RPB and AL from ML. First, terminal labeling in RPB was concentrated in the dorsal half of the field, with sparse inputs to the ventral half (Figures 4A,B). In contrast, the projections to CPB from ML were more uniform from dorsal to ventral across the STG surface, then weakened near the transition to TPO in the STS (Figures 4C–E). The concentration of labeling in the dorsal CPB may reflect topographic gradients, but also may conform to a possible cytoarchitectonic boundary, separating the dorsal and ventral RPB. This possibility has been documented on the basis of architectonic features (Saleem and Logothetis, 2012).Second, in AL, terminals are located in a continuous band involving L1–3a that extended to the lateral edge of the core area, R (Figure 4B). Vertical patches of labeling spanning multiple cell columns were also located in AL (Figure 5D). These were characterized by somewhat higher concentrations of terminals in L4 and L2/3a. Thus, there are inputs to L1 across all of AL with patches characterized by feedforward and lateral inputs.

Altogether, the projections of area ML are consistent with the flow of information along the core-belt-parabelt axis, feeding also into TPO, and also along the caudal-rostral axis within auditory cortex.

#### Projections of CPB

With a few notable exceptions, the principal outputs of the ventral CPB injection were very similar to that of caudal ML, reaching nearly all of the core, belt, parabelt areas along the entire rostral-caudal extent of the AC, as well as TPO and Tpt (Figures 6, 7, 10). The span of the CPB projections covered a larger range along the rostro-caudal axis than those of ML, and therefore a greater number of sections are illustrated at low magnification in Figure 6.

Rostral to the injection site, the main targets of feedforward and lateral projections from the CPB were RPB, RTM, RM, ProA, and TPO (Figures 6A–H). These projections typically formed prominent bands of terminal labeling in L1/2, L4 and L6, with labeling of variable density in other layers. In some locations, most notably TPO, radial columns of anterograde labeling spanning all layers added to these horizontal bands (Figures 7A,C,D,E,H). These columns were separated by zones with reduced label in L3 and L5. Weaker projections to rostral areas reached AL, and primarily L1-3a and L6, and also the core area, R, where inputs were restricted to a continuous band in L1. As observed for area ML, the weakest CPB projections in the rostral direction were to RTL, where only an occasional axon segment was found in L1. No projections were found to the putative core area, RT.

At about the same rostrocaudal level of the injection (Figures 6H–J), strong labeling across all layers formed columns in TPO, CPB, ML, MM, and CL (Figures 7H–K). As in more rostral locations, these dense projections occurred in patches, between which labeling density was significantly reduced. As an example, images of two adjacent patches from area ML are illustrated in (Figures 7H,I). In the left panel (H), terminal labeling spans all layers, and was most dense in L1/2. In the right panel (I), terminal labeling was reduced and mainly found in L1/2 and L6. The visible band in L4 is mainly produced by non-specific background staining, as only a few labeled terminals were found there.

Also at this rostrocaudal level, note that projections to A1 from CPB were limited to L1, which typically formed a continuous band that ran across the entire lateral to medial extent of A1 at all levels (Figure 7L). Combined with similar projections to L1 of R, it appears that this part of CPB projects evenly to L1 across the entire surface of A1 and R in the core. Projections to MM were concentrated in L1–3a and L5–6, but very sparse to absent in L3b–4.

Caudal to the injection site, terminal labeling was present in the belt areas, Tpt, and TPO. In CPB (Figure 7N), terminal labeling was mainly located in L1-3a and L5–6, but very sparse in L3b and 4. Further caudal in CPB, the laminar pattern was maintained, but projection density was reduced (Figure 7O). In CL, dense patches of labeled terminals spanning all layers occurred in patches (Figure 7Q), but also zones of lighter labeling concentrated in L1/2 and L6 were present between patches in CL (Figures 6O). In CM (Figure 7P), terminal labeling was primarily in L1/2 and L6, similar to the lighter zones in CL. In caudal TPO, the patchy columnar labeling seen in rostral TPO and other areas continued across at least the lateral 2/3 of the upper bank (Figure 7K) of the STS before diminishing at the caudal level of Tpt (Figures 6I–P). In Tpt, anterograde projections mainly targeted L1–3a (Figure 7R).

Together, these data indicate that except for RT and RTL, CPB has some type of projection to all areas of the auditory cortex, including the core. Feedforward and/or lateral projections mainly target rostral belt and parabelt areas, TPO, and the lateral belt areas adjacent to CPB. Varieties of feedback projections were more typical of the core and caudal medial belt areas. As noted for ML, these projections are consistent with prominent paths of information flow along the core-belt-parabelt-TPO and caudal-rostral axes in the auditory cortex.

### Case 3: CM injection

In Case 3, a BDA injection was placed into the rostral and medial portion of CM, near its junction with MM (Figures 8E,F). Overall, anterograde and retrograde transport was very strong to the caudal portion of auditory cortex and uniformly weak or absent to rostral areas.

Rostral to the injection site, BDA labeled axons and terminals were found in A1, ML, MM, and CPB, but not in any of the rostral core, belt or parabelt areas. A few labeled somata were noted in the insula of the most rostral section illustrated (Figure 8A, open symbols), at the level of R, but no labeled axons or terminals were found at this level in any field. In rostral A1 and the transition from MM to RM (Figures 8B,C), a small number of axons and terminals were found in L1–3 (not illustrated). Caudally (Figures 8D, 9B,C) labeling in A1 and MM was fairly high in L1, moderate in L2–3b, then light in L4–6. In ML, labeling was found in patchy columns, where terminal labeling was concentrated in L3a–4, and light in L1–2 and L5–6 (Figure 8A). In CPB rostral to the injection site, only a few isolated axons were found scattered in L3.

Caudal to and in line with the injection site in CM, terminal labeling in CL and other portions of CM formed patchy columns that were distributed throughout the territory covered by both fields (Figures 8F,G, 9D,E). In these columnar patches, labeled terminals were found in all layers, but the distribution was uneven. The greatest concentrations of terminals were in L3b and 4, with lighter labeling in L1–3a and weak labeling in L5–6. The patches were typically linked by reduced axon and terminal density in all layers, but a continuous band in L4 remained prominent, visible even at low magnification (e.g., Figure 8G, area CL). Although not entirely visible in Figure 8G, the L4 band was fairly continuous from CM to CL and into CPB on the STG. The terminal labeling in L4 of CPB (Figure 9G) was much lighter compared to the patches in CM and CL, but comparable to labeling in the intervening zones between patches. In Tpt, a few branching axons with labeled terminals were found in L2-3b and L5–6, with a very light band of terminal labeling in L4 (Figure 9H). This laminar pattern was very similar to that observed in CPB caudal to the injection, but with reduced axon density. In caudal TPO (Figure 9F), terminal labeling was concentrated in L4, with sparse labeling in other layers.

Although the spread of projections from CM was more constricted than for ML and CPB, the areal and laminar projection patterns were consistent with information flow along the same major anatomical axes. We are not certain whether the restricted projections, especially in the rostral direction, exemplify the projections of this part of CM, or whether unknown methodological factors limited transport, or perhaps both.

### Laminar projection patterns

The most common laminar patterns observed were: (1) columns of labeled axons and terminals that spanned all layers, but with distinct or prominent bands in L1, L4, and/or L6. This was typical of projections from areas in presumably lower hierarchical stages to one or more areas at a higher stage where feedforward projections were found (e.g., ML and MM to CPB; ML and CPB to RPB and RM; MM, ML and CPB to TPO); (2) terminal labeling focused in supragranular and infragranular layers that avoided the middle layers, including L3b and 4, corresponding to a lateral type of projection that lacked a feedforward component; and (3) terminal labeling concentrated primarily in L1 or L1–2 (Figure 10, right panels). This was typical of projections to A1 and R from CPB and ML, for example, implying a feedback style of input from higher to lower stage of processing. Other examples of this type were from CPB to CM and portions of CL. A somewhat unusual pattern was observed in the projections from CPB and ML to Tpt, which were focused in L1–3a. A very similar pattern characterized the projection from CM to MM and A1, which favored L1–3b. One other unusual pattern was the projection to ProA from ML and CPB, which produced labeled terminals almost entirely confined to L4 and L6. This is the only area that received inputs that did not also at least have inputs to L1 or other supragranular layers.

### Reciprocity and non-reciprocity of connections

Although we did not focus on the patterns of retrograde cell labeling in this study, a few observations are worth noting for future consideration. Most of the locations that received inputs from CPB, ML, or CM also contained retrogradely labeled cells that project back to the injection site. Generally, these labeled somata were located in L3, and less often in L5 or 6. The absence, or near absence, of retrogradely labeled cells was noted in the connections between several areas. Examples included: (1) CPB to A1, R, ProA, and some portions of CM; (2) ML to A1, R, ProA, and Tpt; and (3) MM to TPO, CPB, and Tpt (Supplementary Figure 2 and Supplementary Table 2). Weaker retrograde labeling was also frequently observed in the territory between columns in which dense terminal labeling across layers was accompanied by numerous retrogradely labeled somata concentrated in L3. The lack of labeled somata in these patches, or zones, was most obvious in the projections to TPO where terminals labeled bands in L1/2, L4, and L6 that joined the more prominent columnar patches. These intervening zones or patches are, therefore, sites which receive inputs from the injected areas, but may not project back to those sources.

The absence of labeled neurons in a location may be significant, but the relative rarity of retrogradely labeled cells in L5 and 6 is of questionable validity, as this would imply that most of the reciprocity between connected areas is accomplished via the connections of L3 neurons. This is unlikely. One possible explanation is that this reflects a technical artifact. It is widely known that 10 kDa BDA does not produce extensive retrograde labeling of neurons. Further, we have noted in prior studies of macaque auditory cortex that retrograde transport of the fluoruby and fluoremerald dextran tracers tends to be biased toward supragranular neurons for some unknown reason (Smiley et al., 2007). On one hand, in all of the areas where significant concentrations of terminals were found, retrogradely labeled cells were also present. By that definition, we could surmise that those inter-areal connections were reciprocal. However, the labeled cells were usually concentrated in L3, with fewer cells in L5 or 6, which may be an underrepresentation of the actual projection. We would add here that in contrast to cases 2–3, the CTB injection of ML in Case 1 (Figure 2) produced robust retrograde labeling of supragranular and infragranular neurons in most areas, which provides additional support for the biased transport conjecture. Therefore, although the absence of labeled somata in an area or layer may accurately reflect the absence of a connection, we cannot be entirely certain. For this reason, we elected not to emphasize the retrograde connection patterns in this study. For reference, however, plots and cell counts for Case 2 are summarized in Supplementary Figure 2 and Supplementary Table 2.

## Discussion

The purpose of the present study was to explore the laminar projections of selected caudal belt and parabelt areas, pursuant to a complete survey of the laminar projections of each area of the macaque monkey auditory cortex. Because prior studies and our current models of auditory cortical organization are based primarily on the analysis of neuronal somata labeled by retrograde transport, surprisingly little is known about the laminar circuitry of auditory areas within the superior temporal region. From those foundational earlier studies, low resolution wiring diagrams were generated, which now form the basis of our working models of auditory cortex organization in primates (Hackett et al., 1998; Kaas and Hackett, 1998, 2000; de la Mothe et al., 2006; Hackett, 2011). These diagrams depict connections between areas using lines and arrows (Figure 1), but lack information about the laminar projections of these areas. Therefore, much remains to be learned about these circuits by generating high-resolution wiring diagrams of the laminar circuitry, noting that such models were generated long ago for the visual cortex (Felleman and Van Essen, 1991), and continue to be refined (Markov and Kennedy, 2013). Looking ahead, development of these models is essential for generating and testing meaningful and informed hypotheses about auditory cortical function. Although limited in scope, the present study yielded several new discoveries of sufficient importance to compel modifications of our working model, as discussed below. These small steps increase our motivation to greatly expand this line of inquiry, as additional modifications of the model may result.

To summarize the present findings (Figure 10), the laminar projection patterns of the caudal belt and parabelt support and extend the hypothesis that information flows along two major axes in auditory cortex: core-belt-parabelt and caudal-rostral. First, projections with feedforward characteristics are directed from the caudal belt areas (ML, CM) to caudal domains of the parabelt (CPB) and TPO. The CPB also projects in this manner to caudal TPO. These patterns are consistent with a stream of information flow directed along the core-belt-parabelt axis of auditory cortex, that also feeds strongly into TPO from two different stages of the hierarchy (caudal belt and parabelt). Second, feedforward patterns were also evident in the projections of the caudal belt and parabelt to rostral belt and parabelt areas and rostral TPO. Some of these areas even received overlapping inputs from caudal belt and parabelt (e.g., RPB, RM, and RTM). Overall, these patterns are consistent with a flow of information from caudal to rostral among auditory and related areas in the superior temporal region (Hackett, 2011). Overlaid on these two major patterns of projections were the more complex area-specific projection patterns, for which the laminar relationships were more variable. The balance of the discussion highlights some of the more interesting details, which are presented in the context of a revised model of auditory cortical wiring.

### Similarities and differences in the projections of ML, CM, and CPB

The similarities and differences in the connections of ML, CM, and CPB were enlightening with respect to general patterns of information flow and differences between individual areas. Perhaps the most robust finding was that ML and CPB have comparable laminar patterns of feedforward and lateral projections to several of the same belt and parabelt areas located rostral to or in line with the location of their injections (e.g., *rostral:* RPB, RTM, RM, ProA; *caudal:* CPB, and caudal TPO) (Figure 10). Rostral CM targeted some of the same areas (CPB and caudal TPO). These projections often spanned all layers, but were usually characterized by prominent bands of terminal labeling in L1/2, L4, and L6. Overall, these results indicate that the outputs of ML, CM, and CPB are directed along the two major anatomical axes within auditory cortex (core-belt-parabelt and caudal-rostral). The forward-directed projections along these axes have a prominent serial component that extends into TPO, but the projections to this region are not strictly serial since outputs from both belt and parabelt areas directly reach this field. These connections are discussed further below.

Other similarities were the laminar patterns of the projections that ran against the dominant feedforward gradients. Projections from ML and CPB to areas caudal to their injection sites (MM, CM, CL, caudal CPB, Tpt) were biased toward supra- or supra- and infragranular layers, avoiding the middle layers, reflecting feedback or lateral connectivity. Similarly, projections to the core areas, mainly A1 and R, generally avoided the middle layers and were often concentrated in L1. Similarly, the projections of CPB to most of the belt areas also avoided the middle layers. These patterns indicate that feedback types of projections tend to characterize information moving in the opposite direction along the major axes. Elements of these patterns have been variably noted in prior studies (Galaburda and Pandya, 1983; de la Mothe et al., 2006).

In terms of differences between the injected areas, a few are highlighted here. First, ML had feedforward and lateral projections to AL, but the projections from CPB to AL reached supra- and infragranular layers only. This indicates that AL is in the line of rostrally-directed feedforward projections from ML in the caudal belt, but not from the caudal parabelt. The absence of feedforward inputs to AL from CPB is consistent with straightforward core-belt-parabelt hierarchical relationships (Hackett et al., 1998). A second notable difference was that CPB had dense feedforward and lateral projections to caudal and rostral TPO, but projections to rostral TPO from ML were sparse to absent. This is intriguing since ML had strong forward projections to auditory areas at that rostral level (i.e., RPB, RM, and RTM), but not rostral TPO. It may turn out that CPB is the only caudal area with significant projections to rostral TPO. We predict that some or perhaps all of the rostral belt and parabelt areas will target that area, however. A third difference was that, whereas forward directed projection from the rostral CM injection targeted some of the same areas as ML and CPB (i.e., CPB, caudal TPO), projections were also concentrated in the middle layers of ML, CL, caudal portions of CM, and weakly in gyral Tpt. This suggests that some feedforward projections are directed laterally and caudally from the rostral CM position. Evidence of caudally-directed information flow has been observed in some studies (de la Mothe et al., 2006), but the data remain thin and will require further study of the caudal and medial belt areas. Finally, the absence of projections to all rostral auditory areas from this CM injection was striking. We are not certain whether technical factors could account for this, as projections from this region to rostral locations were noted in marmosets and macaques (de la Mothe et al., 2006; Smiley et al., 2007).

Finally, the concentration of inputs to L1/2, L4, and L6 indicate that, in addition to classic feedforward projections (to L4), significant inputs also terminate in other layers. This implies that within the bundle of projections from one area to another are multiple “strands” that target neurons in different layers. Although highly intriguing, it is not known whether the signals carried along each of these strands bear the same information or even have the same timing. At present, we do not know the specific cell types or laminar positions of the projecting (source) neurons, only that the majority are pyramidal neurons in layers 3 and 5. Future anatomical studies should incorporate methods to dissect these details. Ideally, these would be coupled with physiological recordings using laminar arrays to characterize the properties of the signals carried by each of these strands and their impact on neurons in all layers.

In summary, there were prominent similarities in the feedforward, lateral, and feedback projections of the caudal belt and parabelt areas that were injected. These lend support to our hypotheses about information flow in auditory cortex (Hackett, 2011). The results also reveal significant differences in the laminar projections of individual areas. This highlights the notion that each area, and likely each layer, has a different functional role, and sets the stage for studies that can bring out those features.

### Divergent and convergent projections blur hierarchical relationships

The projection patterns of the caudal belt and parabelt had divergent and convergent characteristics. These patterns were in line with some, but not all, of the hierarchical relationships established in prior studies.

Divergent projections were reflected in two main ways. First, multiple areas of auditory cortex (and TPO) were labeled by injections of each area (Figure 10). The fact that a single area of auditory cortex has connections with several others is not novel, but one worth exploring further, since the present findings indicate that each area can foster feedforward, lateral or feedback projections to several other areas and at more than one level of auditory processing. ML, CM, and CPB had projections to multiple core, belt, and parabelt areas, as well as TPO. Also notable is that none of these tracer injections filled the entire target area. This is interesting since each of the injected areas is large, and its connections could be topographically distributed, meaning that the projections from different loci within a source area may be different in some ways (e.g., tonotopy, binaural integration, inputs from other areas, etc.). It will be important in future studies to compare injections placed in different portions of the same area to reveal whether its outputs are topographically organized. Second, within a single recipient area (e.g., RPB, TPO, A1), the projections from one of the injected areas were typically not contained within a single topographic locus (e.g., point-to-point). Instead, inputs were often distributed over multiple loci with different laminar profiles (e.g., Figure 10, twin panels in CPB, ML, A1, TPOc, CPB, CL). For example, patches that contained columnar labeling across all layers were often separated by inter-patch regions where terminal labeling was concentrated only in L4 and L6. This was common in belt, parabelt, and TPO. In other areas, the inputs were evenly distributed in some layers across much or possibly all of the entire field (e.g., continuous labeling of bands in L1 of A1 and R). These varied patterns of divergence imply that the outputs of a given area are processed in parallel by several areas, and by multiple locations within each of the recipient areas. There is little evidence of point-to-point connectivity in these circuits.

Convergent projections from two or more sites onto a single area or locus within an area were frequently observed. Three key observations are worth noting here. First, the projections of at least two, and sometimes all three, injected areas reached many of the same areas (Figure 10). The main exception, noted above, was rostral TPO, which was only reached by the CPB injection. Second, these projections sometimes originated from different levels of the core-belt-parabelt hierarchy. RPB, RM, RTM, and caudal TPO are examples of recipient areas in which convergent projections originated in belt and parabelt areas. Third, projections to a single area often overlapped in a single column, layer, or layers. An obvious example is in Figure 3, where fluorescent labeling was used to reveal the projections from ML and CPB in the same sections from Case 2. Overlap was substantial in L4 and L6 of CPB and TPO. Although CM was injected in a different case and not reflected in Figure 3, the locations of its projections to L4 of the CPB and TPO (Figures 8, 9) rather strongly suggest that its inputs would also be overlapping. Altogether, these patterns imply that each area, specific layers within each area, and even multiple columns within each area process convergent inputs in parallel from two or more other auditory cortical areas at different hierarchical levels.

The functional implications of such widespread divergence and convergence are not very clear, as there are minimal data on the differences in neuronal response properties between hierarchical areas. Recent neurophysiological data from our laboratory provide some room for speculation. In recordings from 10 core, belt, and parabelt fields, an increasing gradient in response (spike) latencies was observed along the core-belt-parabelt and caudal-to-rostral axes (Camalier et al., 2012) in response to clicks, tones, and noise bursts The gradient was strongest from caudal to rostral areas, and weakest from belt to parabelt. The results of this study, and several others with related findings, generally support the notion that feedforward signal flow is directed along the two major anatomical axes. However, we also noted that mean response latencies were only slightly longer in parabelt vs. lateral belt areas, and that their distributions were highly overlapping. When considered alongside the present study, one could predict that the information delivered to an area, or specific location within an area, by convergent projections from ML and CPB may arrive within a narrow temporal window. This would apply to several areas, based on the present study (RPB, RM, RTM, caudal TPO). Although the nature of the information delivered to a given site through convergent inputs is presumably distinct, those signals could reach that site at about the same time. This is especially intriguing since multiple areas at different hierarchical levels appear to receive at least some convergent inputs from different levels. In addition, for a given site, the precise timing of these events could vary between input layers. As one example, earlier arriving (e.g., modulatory) inputs to L1 could set the tone for later arriving (e.g., driving) inputs to L4, or perhaps reset the phase of ongoing oscillations (Lakatos et al., 2005b).

Along these lines, then, an important future line of inquiry will be to explore the possibility that each of these diverging and converging strands has different functional properties. It is likely that converging inputs from several areas are distinct, but are the divergent projections from one site propagating the same signal in parallel to multiple others? Do those signals differ by the laminar position and cell type of both source and target? These details are essential for understanding the ways in which signals are processed and distributed between areas of auditory cortex.

### Feedback projections to the core from the parabelt

It has been frequently observed in previous studies in primates that the parabelt does not receive significant input from the core areas A1 or R, and sparse inputs from RT. Projections to the parabelt from within the auditory cortex arise almost exclusively from the belt areas. This is the key anatomical support for a core-belt-parabelt hierarchy (Hackett et al., 1998). However, as previously noted in macaques (Pandya and Rosene, 1993) and marmosets (de la Mothe et al., 2006), the parabelt region does appear to have a significant projection back to L1 of the core. In the present study, our results affirm those observations, and also indicate that those inputs are broadly distributed over the core (Figure 10, laminar profiles). The ventral CPB location injected in Case 2 projected evenly to L1 across the entire surface of A1 and R, although input to L1 of the putative third member of the core region, RT, was very sparse. The broad spread of the L1 projection over the core is very similar to that noted by Pandya and Rosene (1993) after a large isotope injection of the STG that probably involved CPB and ML. If we assume this pattern to be true of the parabelt region in its entirety (rostral and caudal divisions), then the feedback projections to the core would form an expansive and dense matrix over the entire core region that lacks obvious rostrocaudal topography. Given that apical dendrites from subpopulations of cells in almost all layers ramify in L1, the projections to L1 from even a single location in the parabelt could exert a powerful influence over global activity within the core region.

### Is the rostral medial belt a connectional crossroads?

In our studies of the connectivity of the auditory cortex in primates, we have noted that the rostromedial belt area, RM, is broadly connected with rostral and caudal auditory areas in a manner distinct from other belt areas (Hackett et al., 1998; de la Mothe et al., 2006; Smiley et al., 2007). See also Galaburda and Pandya (1983) (Figure 10). Retrograde tracing studies showed that whereas the caudal and rostral belt areas tend to have stronger connections with other caudal and rostral areas, the connections of RM appeared to lack such topography. Thus, the outputs of RM are more broadly distributed to belt and parabelt areas along the caudal-rostral axis. The surprising results of the present study add to this quandary, by revealing that the caudal belt and parabelt are sources of strong convergent inputs to RM that have both feedforward and lateral features, such as dense terminal labeling in L4, L6, and other layers. We also noted that RTM received similar inputs from the same areas. In contrast, the caudal medial areas in this study (MM, CM) did not have these convergent input profiles, implying that there is not a generalized pattern of feedforward or lateral inputs to the medial belt from the lateral belt or parabelt.

### Inputs to area tpt

The caudal borders of the belt and parabelt regions are bordered by the temporal parietotemporal area (Tpt), which is mostly known for its multisensory features, including auditory responsiveness in some domains (Leinonen et al., 1980). Systematic studies of the neurophysiological properties have not been achieved so far. In prior anatomical studies from our research groups, retrograde tracer injections of Tpt and adjacent belt areas (CM, CL) revealed that its strongest cortical connections included the caudal belt and parabelt regions, whereas connections with the auditory core region are sparse (Hackett et al., 1998; Smiley et al., 2007). The principal thalamic inputs to Tpt include the medial/magnocellular division of the medial geniculate body (MGm) and multisensory nuclei of the posterior thalamus (i.e., suprageniculate, Sg: limitans, Lim; posterior, Po; medial pulvinar, PM), whereas inputs from the dorsal divisions of the MG (MGd) are sparse and variable (Hackett et al., 2007). On the basis of these connections, we have long considered Tpt to be an auditory-related field that is strongly influenced by the caudal belt and parabelt, and other sensory areas.

In the present study, projections to Tpt from CPB and ML targeted L1–3a, and sparse projections from rostral CM reached L1–4 (Figure 10). In the absence of other data, these patterns raise questions about which, if any, of the auditory cortical areas are a significant source of feedforward inputs to Tpt? The most likely sources would be CL and CM. Although our rostral CM injection revealed only sparse projections to L1–4 of gyral Tpt, perhaps stronger inputs may arise from caudal CM and parts of CL. In the absence of significant feedforward projections to L4 of Tpt, however, it is still possible that the inputs to L1–3a from caudal belt or parabelt areas could significantly impact auditory activity in this area. Given its position in the temporal-parietal-occipital junction, and projections to posterior parietal and dorsal prefrontal cortex (Hackett et al., 1999; Romanski et al., 1999a,b; Lewis and Van Essen, 2000), Tpt is potentially important link between higher order sensory cortex and the targets of the dorsal stream. Detailed studies of Tpt are long overdue.

### Feedforward projections to TPO from caudal belt and parabelt areas

In numerous prior studies, it has been observed that areas of the STG corresponding to the auditory belt and parabelt are broadly connected with areas on the upper bank of the STS corresponding to the TPO. We did not make efforts to subdivide TPO architectonically, but it appears that most of the terminal and cellular labeling was located in the rostral (TPOr), intermediate (TPOi) and caudal (TPOc) divisions (~ TPO2–4), which approximately corresponds to the superior temporal polysensory area (STP) in other nomenclature (Jones and Powell, 1970; Seltzer and Pandya, 1978, 1989b, 1994; Cipolloni and Pandya, 1989; Barnes and Pandya, 1992; Cusick et al., 1995; Seltzer et al., 1996; Hackett et al., 1998; Padberg et al., 2003). These studies, which primarily used retrograde tracers, revealed that populations of labeled cells in TPO are rather dense, tend to be clustered in patches, and exhibit some degree of rostral-caudal topography, although there is also substantial overlap of rostral and caudal areas of the belt and parabelt along this axis. Data on the laminar input patterns from anterograde tracers are unfortunately rather scarce. Seltzer et al. (1996) made a large injection of the caudal parabelt, confined to the surface of the STG, that produced patches of terminal labeling along the caudal-rostral extent of TPO in the upper bank of the STS. In a second case, the CPB injection also extended into the upper bank of the STS. In this case, additional terminal labeling was found to extend beyond TPO and the upper bank to the fundus and lower bank, producing label in areas such as MT, MST, and FST. Cusick et al. (1995) also found that injection of the caudal parabelt produced patches of terminal labeling in TPO. In both studies, it was noted that these terminations spanned across layers in columns, were focused on L4, or were mixed. Thus, the laminar patterns they observed are highly similar to those identified in the present study (Figure 10).

Almost all of the existing data reveal that connections with auditory cortex do not extend significantly beyond the fundus of the STS to its ventral bank or to the inferior temporal gyrus (ITG), nor are there any clear connections with the middle temporal (MT) complex, or V5, which is a visual region known to be involved in visual motion processing. Otherwise, TPO and adjacent fields are broadly connected with primary and secondary sensory areas of visual and somatosensory cortex, and higher order areas of prefrontal and posterior parietal cortex (Seltzer and Pandya, 1978, 1989a,b; Pandya and Seltzer, 1982; Ungerleider and Desimone, 1986; Boussaoud et al., 1990; Cusick et al., 1995; Lewis and Van Essen, 2000; Saleem et al., 2000; Padberg et al., 2003; Markov et al., 2014). The connections of parietal and dorsolateral prefrontal cortex tend to overlap in rostral and caudal sectors of TPO, while connections of the posterior parietal and STG tend to be adjacent and non-overlapping (Barnes and Pandya, 1992; Seltzer et al., 1996). An interesting feature of the convergence of inputs in TPO is that they can be patchy, overlapping or interdigitating. It is not yet clear how patches associated with auditory cortex relate to those associated with other cortical fields. Although unimodal, bimodal, and trimodal responses to auditory, somatosensory, and visual stimuli have been recorded in TPO (Benevento et al., 1977; Desimone and Gross, 1979; Bruce et al., 1981; Baylis et al., 1987; Hikosaka et al., 1988; Schroeder and Foxe, 2002), the anatomical data suggest that while sensory inputs to STS may be initially segregated by modality, local connectivity provides a basis for multisensory interactions.

### Connections beyond the superior temporal region

We did not evaluate the projections to subcortical and other cortical regions for the present study, but in cursory inspections, we did note that the areas injected produced labeling in frontal, medial temporal, and thalamic locations. We did not observe labeling in posterior parietal areas, however, as might have been expected from prior studies (Pandya and Kuypers, 1969; Pandya et al., 1969; Lewis and Van Essen, 2000; Smiley et al., 2007). Because transport was judged to be very good from at least two of these injections, we are inclined to conclude that ML and CPB do not have significant projections to posterior parietal areas. Rather, judging from the earlier studies and more recent data (Markov et al., 2014), it is likely that Tpt, and perhaps CM or CL may be the most dominant sources of inputs to posterior parietal areas from superior temporal cortex. Interestingly, one recent study found significant projections between posterior parietal and RPB areas (Markov et al., 2014), suggesting that a more determined survey of these connections is warranted.

### Integration with prior studies

One of the earliest studies of the laminar patterns of projections in auditory cortex was conducted in owl monkeys. Fitzpatrick and Imig (1980) analyzed projections in the core and belt after placing isotope injections into A1 or R. They found that projections from these core areas to belt areas often spanned layers, but sometimes with concentrations in L4 alone, L3a/4, L3a/4/6. These laminar patterns were similar to those observed in the feedforward and lateral projections between areas in the present study. They also found that within the core, projections from A1 to R targeted L4, consistent with a rostrally-directed flow of information within the core.

Three studies used tracers with anterograde or mixed anterograde and retrograde tracing properties to study auditory cortical connections in marmosets (Aitkin et al., 1988; de la Mothe et al., 2006; de la Mothe et al., 2012). Both sets of studies found that projections from core to belt areas often resulted in columnar terminations spanning layers, but often with a focal band of higher density in L4. In de la Mothe et al. (2006), injections of RM and CM or MM labeled columns of terminals in the lateral belt and parabelt areas, variably punctuated by more intense bands in L2/3a, 4 and 6. These columns were often separated by columns of weaker labeling, but it was common for dense terminal labeling to persist continuously in the L4 and 6 bands. This suggested that the medial belt areas give rise to feedforward and lateral projections to the lateral belt and parabelt. One additional note, when anterograde terminals were concentrated in L4 of another belt or parabelt area from these injections, they tended to be located in L4 in sites rostral to the injection site, but not caudal. So, in addition to a core-belt-parabelt pattern, there were hints of a rostrally-directed bias in the feedforward projections from the medial belt.

In their foundational study, Galaburda and Pandya (1983) used isotope tracers with anterograde transport properties to study the connections of auditory areas in the macaque monkey STG. Surprisingly, after 30 years, this study stands alone as the most extensive survey of anterograde projections in the auditory cortex of macaques. A major conclusion of that study supported a rostrally-directed pattern of connectivity between areas corresponding to the core, belt, and parabelt regions (terminology transposed to match our nomenclature). These patterns were tied to progressive stages of architectonic differentiation along this axis (caudal to rostral). Although injection sites were typically large, covering more than one field, some general patterns were identified that were also observed and refined by the present study. For example, in case IX, a large isotope injection involving the caudal parabelt and Tpt resulted in feedforward projections to the caudal parabelt and lateral belt, projections to L1 of the core (A1), and terminations across layers in areas corresponding to the RM and MM fields. In cases IV and V, injections of primarily the RPB generated feedforward projections to belt and parabelt areas rostral to the injection site, but predominately L1 projection to caudal CPB. In general, the patterns showed that rostrally-directed projections mainly originate in L3 and terminate in L4 or across all layers in the rostral targets. Caudally-directed projections tended to originate in infragranular layers and terminate in superficial layers. Projections from the core to belt areas were focused on L4, and projections from belt and parabelt to core were focused in L1. Projections from lateral belt to medial belt were spread broadly across layers. Overall, these patterns were generally comparable to those of the present study, although the detailed laminar patterns of connectivity revealed herein varied in a more specific manner between aerial targets, and the greater sensitivity of the tracers revealed the presence of axons and terminals in additional layers.

### Caveats and future directions

The present study is based on only 4 tracer injections in 3 areas of 3 different experimental cases. Ideally, we would aim to have at least two injections from each target area as a means to evaluate reliability. There is always some variability in transport between injections into cortex. Reliable control of the precise size and location of the injections is generally not possible, even if all experimental variables are exactly repeated. In part, this is because the transport properties of different tracers vary, and the uptake and transport of the same tracer can vary due to factors that appear to be beyond experimental control. For the cases illustrated here, the injections were judged to be very good in terms of placement within a single area, involvement of all layers, and transport, and so we have a high degree of confidence in the results. Clearly, additional studies of this type are desperately needed to obtain detailed and comprehensive wiring diagrams of the projections of all auditory cortical areas, and bolster the findings of the present study. This will be a challenging pursuit, as the territory is relatively large, and many of the areas are buried in locations that require passing through or retracting the cortex of intervening regions. This is a necessary endeavor, however, as our understanding of auditory cortical function depends critically on knowledge of its circuitry. In the meantime, however, the results of this study raised several important questions about the diversity of ways in which signals are passed between areas, layers, and even specific cell types. Many of these questions can be addressed now by using laminar array recording techniques to document the properties of the signals carried by the various strands of projections that reach a given site.

### Conflict of interest statement

The authors declare that the research was conducted in the absence of any commercial or financial relationships that could be construed as a potential conflict of interest.

## Supplementary material

Supplementary Figure 1 **Examples of architectonic features used to delineate areas and layers of auditory cortex**. **(A)** Coronal section at the level of mid-A1 reacted for NeuN IHC (blue fluorescence). Borders between other areas indicated by lines following radial orientation of cell columns. **(B)** Coronal section in same series as the section in **(A)**, but stained for VGluT2. **(C–F)** Coronal section through area ML, showing triple fluorescent labeling of FR, BDA, and NeuN. **(F)** is a grayscale conversion of **(E)**, showing position of layers. **(G–J)** Coronal section through MM. Same conventions as **(C–F)**. **(K–N)** Coronal section through area TPO, showing BDA transport and NeuN. Scale bars: **(A–N)**, 500 μm.

Supplementary Figure 2 **Plots of retrogradely labeled cells in Case 2 following tracer injections into ML (FR, open triangles) and CPB (BDA, filled circles)**.

Supplementary Table 1 **Gray Level Index (GLI) values of anterograde labeling in auditory cortical areas from tracer injections in Cases 2 and 3**. Measurements were taken from the sections illustrated in Figures 4–9. For each injection, GLI values are sorted by area and layer. Letters A–R in the top row correspond to panel numbers in Figures 5, 7, 9. These data are summarized graphically in Figure 10.

Supplementary Table 2 **Retrograde-labeled cell counts and percent of total cells from tracer injections into ML (FR) and CPB (BDA) (Case 2)**. Sorted by cortical area in supragranular (S) and infragranular (I) layers. The results are graphically summarized in the charts below and in Supplementary Figure 2.

## Abbreviations

A1: Auditory area 1 (core)
AChE: Acetylcholinesterase
AL: Anterolateral area (belt)
AS: Arcuate sulcus
Cis: Circular sulcus
CL: Caudolateral area (belt)
CM: Caudomedial area (belt)
CO: Cytochrome oxidase
CPB: Caudal parabelt area (parabelt)
CS: Central sulcus
Id: Insula, dysgranular
Ig: Insula, granular
IPS: Intraparietal sulcus
ITG: Inferior temporal gyrus
Lim: Limitans nucleus
LS: Lateral sulcus
LuS: Lunate sulcus
MGad: Medial geniculate complex, anterodorsal division
MGC: Medial geniculate complex
MGd: Medial geniculate complex, dorsal division
MGm: Medial geniculate complex, magnocellular division
MGpd: Medial geniculate complex, posterodorsal division
MGv: Medial geniculate complex, ventral division
ML: Middle lateral area (belt)
MT: Middle temporal area
Pro: Proisocortical area
proA: Prokoniocortex area
PS: Principal sulcus
PV: Parvalbumin
R: Rostral area (core)
Ri: Retroinsular area
RM: Rostromedial area (belt)
RPB: Rostral parabelt area (parabelt)
RT: Rostrotemporal area (core)
RTL: Rostrotemporal lateral area (belt)
RTM: Rostrotemporal medial area (belt)
S2: Somatosensory area 2
Sg: Suprageniculate nucleus
STG: Supeior temporal gyrus
STS: Superior temporal sulcus
TPO: Temporal parietal occipital area
TPOc: TPO, caudal sector
TPOr: TPO, rostral sector
Tpt: Temporal parietotemporal area
VGluT1: Vesicular glutamate transporter 1
VGluT2: Vesicular glutamate transporter 2.
