# Supplementary figures and images for: Feedforward and feedback projections of caudal belt and parabelt areas of auditory cortex: refining the hierarchical model
Source: Front Neurosci. 2014 Apr 22;8:72. doi: 10.3389/fnins.2014.00072 (PMC4001064; doi:10.3389/fnins.2014.00072)

Supplementary Figure 1

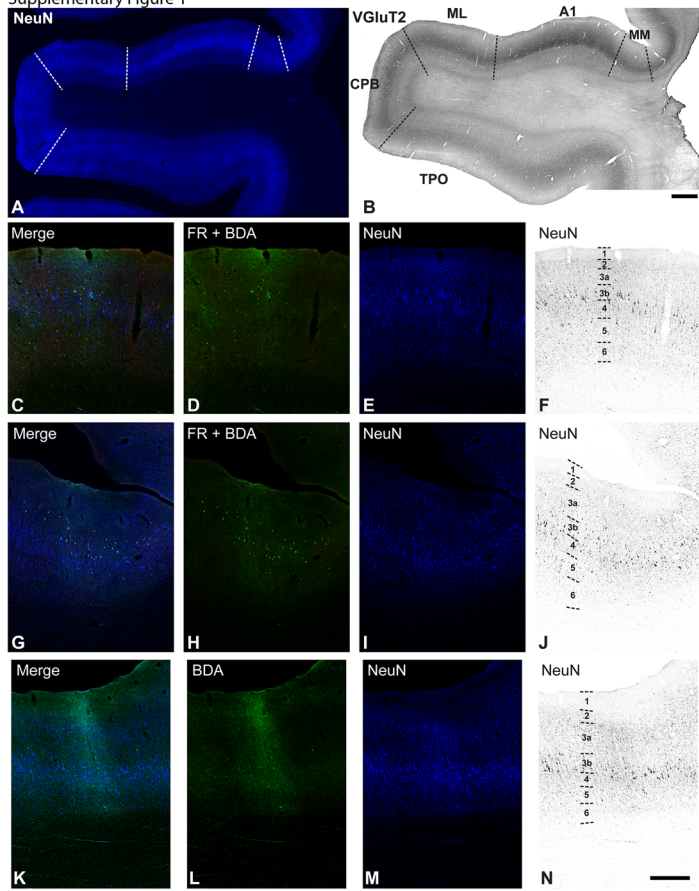

Supplement: Supplementary Figure 1 — Examples of architectonic features used to delineate areas and layers of auditory cortex. (A) Coronal section at the level of mid-A1 reacted for NeuN IHC (blue fluorescence). Borders between other areas indicated by lines following radial orientation of cell columns. (B) Coronal section in same series as the section in (A), but stained for VGluT2. (C–F) Coronal section through area ML, showing triple fluorescent labeling of FR, BDA, and NeuN. (F) is a grayscale conversion of (E), showing position of layers. (G–J) Coronal section through MM. Same conventions as (C–F). (K–N) Coronal section through area TPO, showing BDA transport and NeuN. Scale bars: (A–N), 500 μm. [file Presentation1.PDF]

Supplementary Figure 2

Rostral

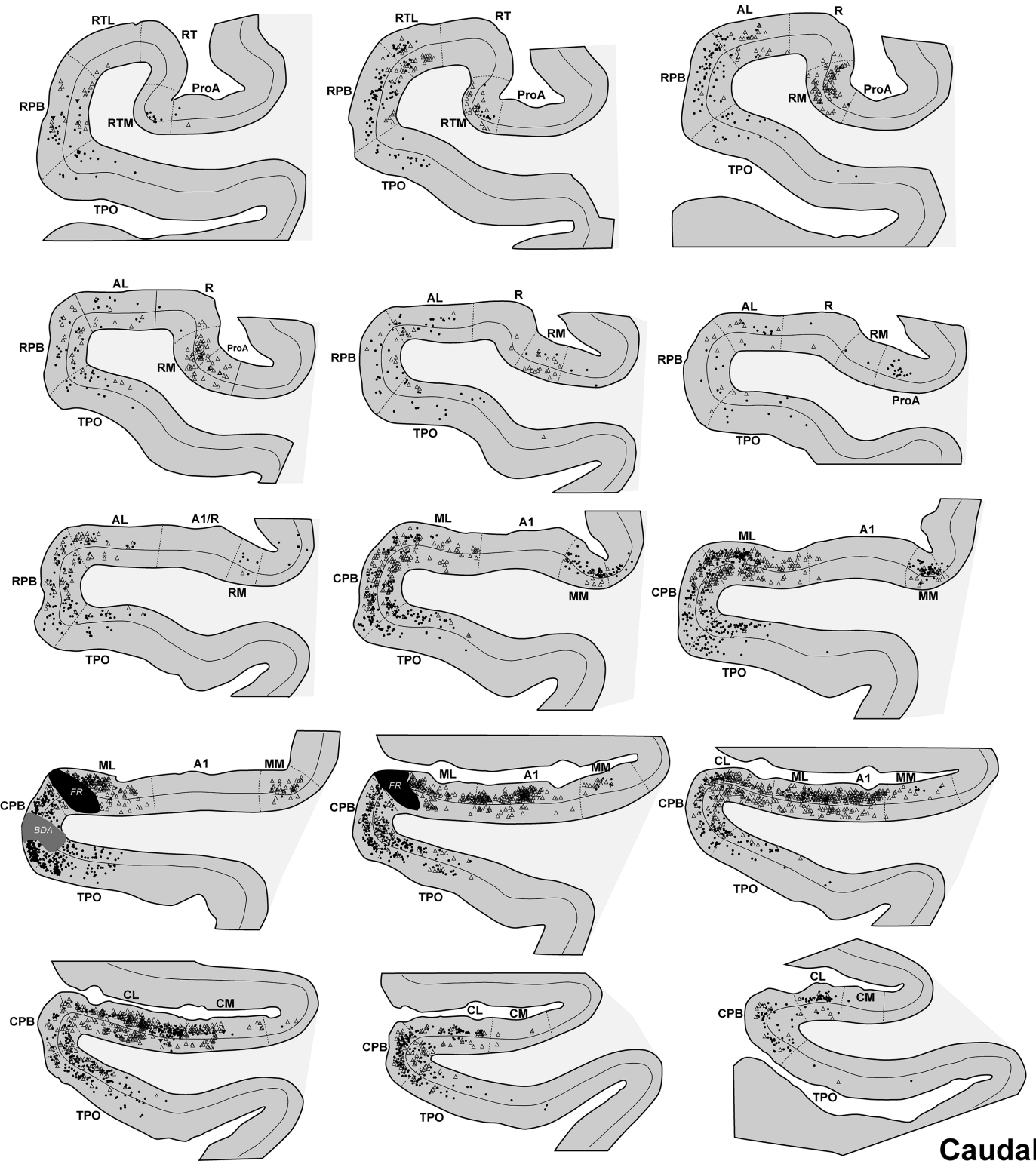

Caudal

Supplement: Supplementary Figure 2 — Plots of retrogradely labeled cells in Case 2 following tracer injections into ML (FR, open triangles) and CPB (BDA, filled circles). [file Presentation2.PDF]
