# Supplementary material for: Feedforward and feedback projections of caudal belt and parabelt areas of auditory cortex: refining the hierarchical model
Source: Front Neurosci. 2014 Apr 22;8:72. doi: 10.3389/fnins.2014.00072 (PMC4001064; doi:10.3389/fnins.2014.00072)
Supplement: Supplementary Table 1 — Gray Level Index (GLI) values of anterograde labeling in auditory cortical areas from tracer injections in Cases 2 and 3. Measurements were taken from the sections illustrated in Figures 4–9. For each injection, GLI values are sorted by area and layer. Letters A–R in the top row correspond to panel numbers in Figures 5, 7, 9. These data are summarized graphically in Figure 10. [file DataSheet1.PDF]

Supplementary Table 1. Gray Level Index (GLI) values of anterograde labeling in auditory cortical areas from tracer injections in Cases 2 and 3. Measurements were taken from the sections illustrated in Figures 4 – 9. For each injection, GLI values are sorted by area and layer. Letters A – R in the top row correspond to panel numbers in Figures 5, 7, and 9. These data are summarized graphically in Figure 10.

GLI index: FR Injection of area ML (Figure 5)

Layer	(A) RPB	(B) RT	(C) RTM	(D) AL	(E) R	(F) RM	(G) CPBa	(H) TPOa	(I) ML
**1**	9	2	13	10	1	14	39	13	39
**2**	10	3	9	9	3	13	54	8	39
**3a**	16	4	11	8	3	13	54	5	32
**3b**	28	4	16	7	2	10	55	8	24
**4**	45	4	27	13	1	12	63	14	24
**5**	15	3	15	7	1	11	28	4	33
**6**	14	0	13	7	1	16	35	11	33

!

Layer	(J) ML	(K) A1a	(L) CPBb	(M) A1b	(N) MM	(O) A1c	(P) TPOb	(Q) CL	(R) CM
**1**	21	14	18	18	18	25	19	43	20
**2**	20	7	20	8	10	16	19	36	13
**3a**	13	3	23	5	10	12	18	33	12
**3b**	9	3	21	3	6	8	15	29	10
**4**	5	3	21	2	3	2	24	22	4
**5**	8	1	10	5	10	4	17	20	6
**6**	9	7	13	5	9	5	19	18	6

!

GLI index: BDA injection of area CPB (Figure 7)

Layer	(A) RTM	(B) ProA	(C) RPB	(D) TPOa	(E) RM	(F) R	(G) AL	(H) MLa	(I) MLb
**1**	11	10	19	9	25	5	9	62	13
**2**	9	9	22	11	21	1	7	54	13
**3a**	23	10	18	14	17	1	6	48	9
**3b**	21	12	22	11	16	1	4	42	5
**4**	26	18	35	26	25	2	2	34	6
**5**	19	7	20	8	15	0	-1	27	5
**6**	19	8	25	17	9	0	-1	16	11

Layer	(J) MM	(K) TPOb	(L) A1	(M) TPOc	(N) CPBa	(O) CPBb	(P) CM	(Q) CL	(R) Tpt
**1**	23	1	8	36	38	21	9	26	33
**2**	18	6	2	43	30	11	4	24	22
**3a**	22	9	1	60	23	12	3	23	15
**3b**	16	10	1	62	24	11	2	26	6
**4**	11	5	2	61	28	10	2	28	3
**5**	18	5	0	48	32	13	1	20	2
**6**	20	6	3	34	30	11	1	27	2

GLI index: BDA injection of area CM (Figure 9)

Layer	A-ML	B-A1	C-MM	D-CM	E-CL	F-TPO	G-CPB	H-Tpt
**1**	23	25	24	18	19	8	4	1
**2**	16	18	17	13	19	8	4	2
**3a**	20	16	15	17	22	8	4	1
**3b**	23	11	14	24	32	6	3	3
**4**	23	8	9	31	37	18	7	3
**5**	9	8	5	12	15	3	1	1
**6**	4	4	3	5	9	2	1	1
