# Supplementary material for: Feedforward and feedback projections of caudal belt and parabelt areas of auditory cortex: refining the hierarchical model
Source: Front Neurosci. 2014 Apr 22;8:72. doi: 10.3389/fnins.2014.00072 (PMC4001064; doi:10.3389/fnins.2014.00072)

Supplementary Table 2. Retrograde-labeled cell counts and percent of total cells from tracer injections into ML (FR) and CPB (BDA)(Case 2). Sorted by cortical area in supragranular (S) and infragranular (I) layers). The results are graphically summarized in the charts below and in Supplementary Figure 2.

	**A1**				**R**				**RT**				**CM**				**MM**			
	FR		*BDA*		*FR*		*BDA*		*FR*		*BDA*		*FR*		*BDA*		*FR*		*BDA*	
	**S**	**I**	**S**	**I**	**S**	**I**	**S**	**I**	**S**	**I**	**S**	**I**	**S**	**I**	**S**	**I**	**S**	**I**	**S**	**I**
**Count**	273	54	1	0	5	4	2	1	0	0	0	0	45	15	15	1	69	18	85	10
**%**	7	1	0	0	0	0	0	0	0	0	0	0	0	0	0	0	2	0	2	0

	**RM**				**RTM**				**ProA**				**CL**				**ML**			
	FR		*BDA*		*FR*		*BDA*		*FR*		*BDA*		*FR*		*BDA*		*FR*		*BDA*	
	**S**	**I**	**S**	**I**	**S**	**I**	**S**	**I**	**S**	**I**	**S**	**I**	**S**	**I**	**S**	**I**	**S**	**I**	**S**	**I**
**Count**	70	46	14	1	13	7	0	12	13	6	18	1	87	63	83	15	363	141	160	8
**%**	2	1	0	0	0	0	0	0	0	0	0	0	2	2	2	0	10	4	4	0

	**AL**				**RTL**				**CPB**				**RPB**				**TPO**			
	FR		*BDA*		*FR*		*BDA*		*FR*		*BDA*		*FR*		*BDA*		*FR*		*BDA*	
	**S**	**I**	**S**	**I**	**S**	**I**	**S**	**I**	**S**	**I**	**S**	**I**	**S**	**I**	**S**	**I**	**S**	**I**	**S**	**I**
**Count**	40	28	50	13	5	17	19	5	169	136	429	167	39	53	156	99	19	49	264	265
**%**	1	1	1	0	0	0	1	0	5	4	12	5	1	1	4	3	1	1	7	7

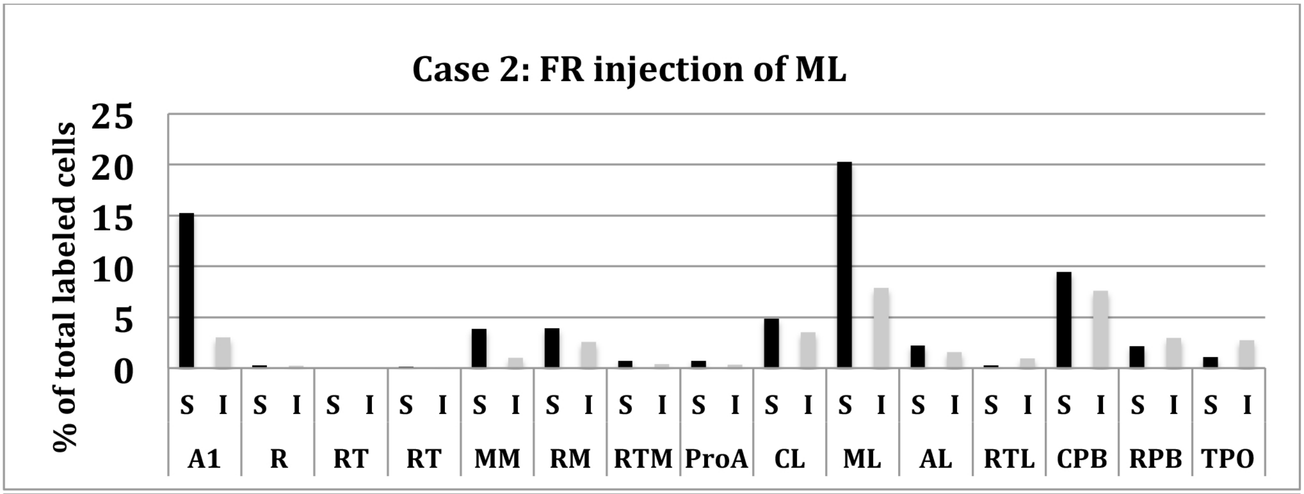

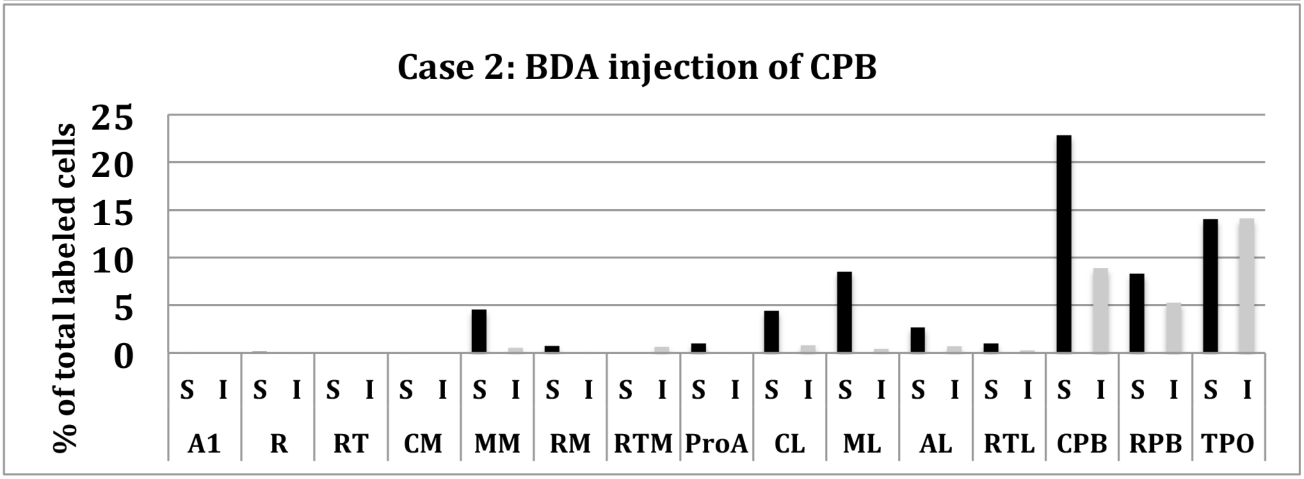

Supplement: Supplementary Table 2 — Retrograde-labeled cell counts and percent of total cells from tracer injections into ML (FR) and CPB (BDA) (Case 2). Sorted by cortical area in supragranular (S) and infragranular (I) layers. The results are graphically summarized in the charts below and in Supplementary Figure 2. [file DataSheet2.PDF]
